# INPP5F translocates into cytoplasm and interacts with ASPH to promote tumor growth in hepatocellular carcinoma

**DOI:** 10.1186/s13046-021-02216-x

**Published:** 2022-01-07

**Authors:** Qianlei Zhou, Jianhong Lin, Yongcong Yan, Shiyu Meng, Hao Liao, Ruibin Chen, Gui He, Yue Zhu, Chuanchao He, Kai Mao, Jie Wang, Jianlong Zhang, Zhenyu Zhou, Zhiyu Xiao

**Affiliations:** 1 Department of Hepatobiliary Surgery, Sun Yat-Sen Memorial Hospital, Sun Yat-Sen University, Guangzhou, 510120 China; 2 Guangdong Province Key laboratory of Malignant Tumor Epigenetics and Gene Regulation, Sun Yat-Sen Memorial Hospital, Sun Yat-Sen University, Guangzhou, 510120 China; 3 Department of Thyroid Surgery, Sun Yat-Sen Memorial Hospital, Sun Yat-Sen University, Guangzhou, 510120 China; 4 Department of Anesthesiology, Sun Yat-Sen Memorial Hospital, Sun Yat-Sen University, Guangzhou, 510120 China; 5 Cellular & Molecular Diagnostics Center, Sun Yat-Sen Memorial Hospital, Sun Yat-Sen University, Guangzhou, 510120 China

**Keywords:** HCC, INPP5F, ASPH, Notch signaling pathway, Nuclear-cytoplasmic shuttling

## Abstract

**Background:**

Increasing evidence has suggested inositol polyphosphate 5-phosphatase family contributes to tumorigenesis and tumor progression. However, the role of INPP5F in hepatocellular carcinoma (HCC) and its underlying mechanisms is unclear.

**Methods:**

The expression of INPP5F in HCC was analyzed in public databases and our clinical specimens. The biological functions of INPP5F were investigated in vitro and vivo. The molecular mechanism of INPP5F in regulating tumor growth were studied by transcriptome-sequencing analysis, mass spectrometry analysis, immunoprecipitation assay and immunofluorescence assay.

**Results:**

High expression of INPP5F was found in HCC tissues and was associated with poor prognosis in HCC patients. Overexpression of INPP5F promoted HCC cell proliferation, and vice versa. Knockdown of INPP5F suppressed tumor growth in vivo. Results from transcriptome-sequencing analysis showed INPP5F not only regulated a series of cell cycle related genes expression (c-MYC and cyclin E1), but also promoted many aerobic glycolysis related genes expression. Further studies confirmed that INPP5F could enhance lactate production and glucose consumption in HCC cell. Mechanistically, INPP5F activated Notch signaling pathway and upregulated c-MYC and cyclin E1 in HCC via interacting with ASPH. Interestingly, INPP5F was commonly nuclear-located in cells of adjacent non-tumor tissues, while in HCC, cytoplasm-located was more common. LMB (nuclear export inhibitor) treatment restricted INPP5F in nucleus and was associated with inhibition of Notch signaling and cell proliferation. Sequence of nuclear localization signals (NLSs) and nuclear export signals (NESs) in INPP5F aminoacidic sequence were then identified. Alteration of the NLSs or NESs influenced the localization of INPP5F and the expression of its downstream molecules. Furthermore, we found INPP5F interacted with both exportin and importin through NESs and NLSs, respectively, but the interaction with exportin was stronger, leading to cytoplasmic localization of INPP5F in HCC.

**Conclusion:**

These findings indicate that INPP5F functions as an oncogene in HCC via a translocation mechanism and activating ASPH-mediated Notch signaling pathway. INPP5F may serve as a potential therapeutic target for HCC patients.

**Supplementary Information:**

The online version contains supplementary material available at 10.1186/s13046-021-02216-x.

## Background

Hepatocellular carcinoma (HCC) is the sixth-most common cancer worldwide and the third-leading cause of cancer-related deaths [1]. The incidence and mortality rates for HCC are still increasing [2]. However, the effect of current therapeutic approaches remains to be satisfied. Understanding the molecular mechanisms of HCC tumorigenesis and progression may help to improve the therapeutic outcomes for HCC patients.

Inositol polyphosphate 5-phosphatases are a large family of enzymes, which are involved in regulating phosphorylation of phosphoinositide and associated with in a series of human pathologies such as the Lowe syndrome, the Joubert and MORM syndromes and several type of cancers [3–7]. Inositol polyphosphate 5-phosphatases contain 10 different isoenzymes and several splice variants in the human genome. Inositol polyphosphate-5-phosphatase F (INPP5F, also known as Sac 2) is a member of inositol polyphosphate 5-phosphatases, which has been demonstrated to hydrolyze different type of phosphoinositide and exhibit different functions, such as regulating endocytic recycling [8], attenuating heart hypotrophy [9], participating in the onset of Parkinson’s disease [10]. Recently, INPP5F is also reported to play an important role in the occurrence and progression of malignant tumor, although the role is inconsistent. In gliomas, downregulation of INPP5F may lead to gliomagenesis [11]. While in chronic lymphocytic leukemia, overexpression of INPP5F is associated with chemoresistance [12, 13].

So far, the clinical significance of INPP5F and its role in HCC is unclear. In this study, we sought to assess the expression and clinical significance of INPP5F in HCC patients, as well as explore the underlying mechanisms of how INPP5F functions in HCC.

## Methods and materials

### Patients and clinical samples

Eighty-eight HCC samples were collected for determination of mRNA levels of INPP5F from Sun Yat-Sen Memorial hospital (SYSMH). The samples were obtained from the HCC patients who undergone hepatic resection between Mar 2015 and Feb 2016. These sample were frozen and stored in liquid nitrogen until further analysis. Six cases of HCC for western blot were collected in Oct 2020 at SYSMH. In addition, a cohort of 232 paraffin-embedded HCC cases diagnosed between Jan 2010 to Dec 2013 at SYSMH was recruited. The HCC and adjacent non-tumor of these patients were collected immediately after surgery, stored in formalin and then made into tissue microarray. The patients’ clinical and prognostic data were acquired from the specimen library of Department of hepatobiliary surgery (SYSMH). There was no relation between RNA samples and paraffin-embedded cases. Histological examination was used to confirm the diagnosis in all patients. None of the patients had received radiotherapy or chemotherapy before surgery. The study protocol was approved by the Ethics Committee of SYSMH. Informed consent was obtained from each patient.

### Immunohistochemistry (IHC)

The tissue microarray was subjected to deparaffinization and dehydration. After antigen retrieval, H_2_O_2_ treatment and non-specific antigens blocking, the slides were incubated with monoclonal mouse anti-human INPP5F (1:200, Abcam, ab236391) at 4 °C. After overnight incubation, the slides were incubated with secondary antibody, followed by DAB staining. The expression levels were scored as previously describe [14]. The total scoring of the tissue microarray was independently completed by two pathologists who had no knowledge of the patients’ clinical data. We defined the case as high expression if the total score was greater than 4 points, otherwise defined it as low expression. Other antibodies used for IHC staining included those against the proteins Ki-67 (1:200, ab16667, Abcam, UK), c-MYC (1:500, 67,447–1-Ig, Proteintech, USA) and cyclin E1 (1:200, 11,554–1-AP, Proteintech, USA).

### Glucose consumption and lactate production assays

Cells were cultured to 40% confluency and then changed with fresh culture medium. After 24 h, the culture medium was collected, and measurement of glucose consumption and lactate production was performed using kits from Biovision (USA, catalogue nos. ab136955 and ab65331) according to the manufacturer’s instructions.

### Animal model

For xenograft model, male BALB/c nude mice aged 3–4 weeks were randomized into two groups (*n* = 6). 5 × 10^6^ MHCC-97H cells stably expressing luciferase and control or INPP5F shRNA were subcutaneously injected into the left flank of these mice. Four weeks later, mice were monitored by bioluminescence with the IVIS imagining system (Xenogen, MA, USA). And then the mice were sacrificed, tumor weight and size were measured. Volumes were calculated using the following formula: Volume (mm3) = [width2 (mm2) × length (mm)]/2. To further detect the effect of INPP5F on tumor growth, negative control or INPP5F shRNA MHCC-97H cells (2 × 10^6^) were orthotopically injected under the liver capsular of NOD/SCID mice (three mice per group). Mice were monitored using the IVIS200 imaging system. All animal procedures were in accordance with the National Institutes of Health guide for the care and use of laboratory animals and approved by the Animal Ethical and Welfare Committee of Sun Yat-Sen University.

### Co-immunoprecipitation assay and mass spectrometry

FLAG-INPP5F and HA-ASPH plasmids were transfected into Huh7 cells. Crude cell lysate was prepared 72 h after transfection. The protein complex interacting with FLAG-INPP5F was obtained using PierceTM Co-Immunoprecipitation Kit (Thermo Fisher Scientific, MA, USA) according to the manufacturer’s instructions. Mass spectrometry analysis of immunoprecipitant was performed by the Medical Research Center of SYSMH. Co-immunoprecipitation and western blot were used for validating the interacted protein identified by mass spectrometry analysis.

### Construction of mutation and truncations

NLSs and NESs in INPP5F aminoacidic sequence were predicted by bioinformatic tools (NLS: http://nls-mapper.iab.keio.ac.jp/cgi-bin/NLS_Mapper_form.cgi, NES: http://www.cbs.dtu.dk/services/NetNES). When constructing the NESs mutation, we changed the leucine in the predicted NESs to alanine in order to make INPP5F lose the nuclear export ability. The three truncations were constructed through deletion of different lengths of aminoacidic sequence in the predicted NLSs to facilitate INPP5F transport out of the nuclear. The mutation and truncation sequences were cloned into the plasmids pcDNA3.1 (Igebio company, Guangzhou, China).

### Statistical analysis

All data analysis was performed using SPSS version 25.0. Student’s t test and Chi-square test were used to analyze quantitative data and categorical data, respectively. Kaplan–Meier analyses and log-rank test were used for survival analysis. The cox proportional hazards regression model was used to verify the independent risk factors based on the variables selected in univariate and multivariate analysis. *P* value < 0.05 were considered to be statistically significant.

## Results

### Increased INPP5F expression predicts poor clinical outcome in HCC patients

**Fig. 1:**
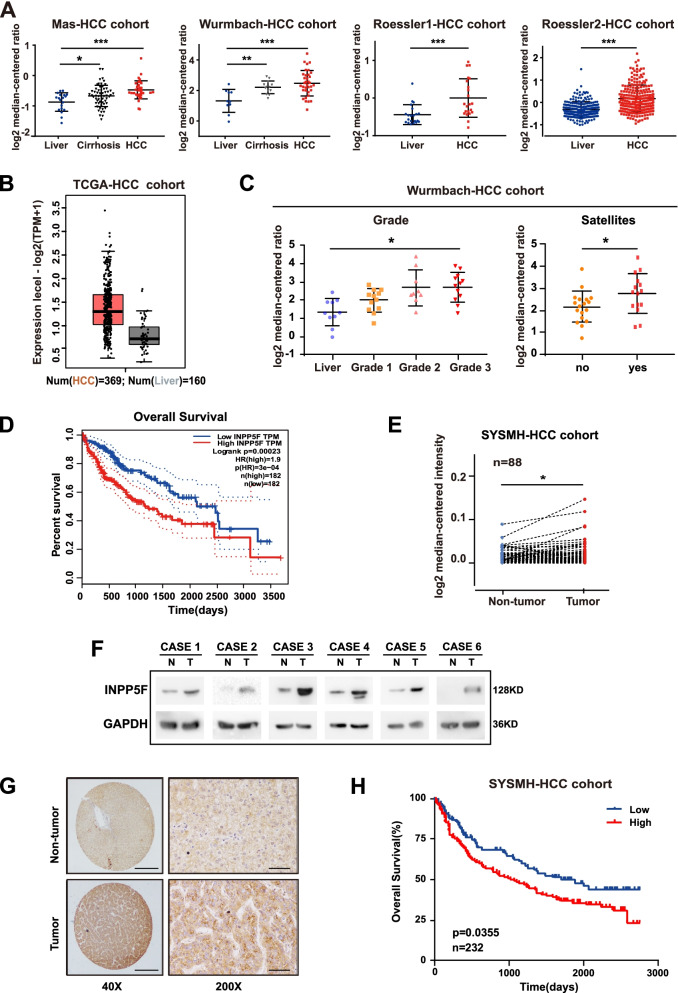
INPP5F expression is elevated in HCC and associated with poor clinical outcome. **A** HCC datasets from Oncomine database (https://www.oncomine.org) showed increase of INPP5F mRNA in HCC samples. **B** Analysis of TCGA-HCC dataset (http://gepia.cancer-pku.cn) confirmed the increase of INPP5F mRNA in HCC samples. **C** Subgroups analysis of the Wurmbach-HCC cohort (https://www.oncomine.org) exhibitd higher mRNA expression level of INPP5F in HCC patients with worse histological differentiation grade and satellite lesions. **D** Kaplan-Meier analysis of the overall survival using the TCGA-LIHC cohort according to INPP5F expression . **E** The mRNA levels of INPP5F in 88 pairs of HCC and adjacent non-tumor tissues were measured by QRT-PCR. **F** The protein levels of INPP5F in 6 pairs of HCC and adjacent non-tumor tissues were measured by Western blot. **G** The expression of INPP5F in 232 pairs of HCC and adjacent non-tumor tissues was detected by IHC. **H** The clinical significance of INPP5F expression in overall survival was confirmed in SYSMH-HCC cohort by Kaplan-Meier survival analysis. ALL *P < 0.05, **P < 0.01, ***P < 0.001. Scale bar: 100 um

To investigate the potential role of INPP5F in human HCC pathogenesis, we firstly employed public databases to evaluate the expression of INPP5F in HCC. Data derived from Oncomine database showed that INPP5F is commonly upregulated in HCC tissues (Fig. 1A). Higher INPP5F expression in HCC tissues is also observed in TCGA-LIHC cohort (Fig. 1B) as well as datasets from GEO database (Fig. S1A). Notably, in the Wurmbach-HCC cohort, higher INPP5F expression level is found in HCC cases with worse histological differentiation grade and satellite lesions (Fig. 1C). Moreover, TCGA-LIHC cohorts showed that as the tumor status and grade increased, an increased tendency for INPP5F expression is observed (Fig. S1B). Kaplan-Meier analysis using the TCGA-LIHC cohort revealed that patients with higher INPP5F expression exhibit a relatively poorer overall survival (Fig. 1D). We further validated these results using data from our center. QRT-PCR and western blot results revealed that mRNA and protein level of INPP5F was frequently upregulated in HCC tissues compared with the corresponding adjacent non-tumor tissues (Fig. 1E and F). IHC staining in 232 pairs of HCC specimens showed consistent results (Fig. 1G). Further analysis showed that high expression of INPP5F was associated with large tumor size, poor tumor differentiation and cirrhosis (Table S1). Patients with high INPP5F expression had shorter overall survival than those with low INPP5F expression (Fig. S1C and Fig. 1H). Multivariate analyses using Cox regression model revealed INPP5F as an independent prognostic factor for overall survival in HCC patients (Table S2). Taken together, data from public databases and our center suggest the oncogenic role of INPP5F in HCC, prompting us to further investigate the role of INPP5F in HCC.

### INPP5F promotes HCC cell proliferation in vitro and in vivo

**Fig. 2:**
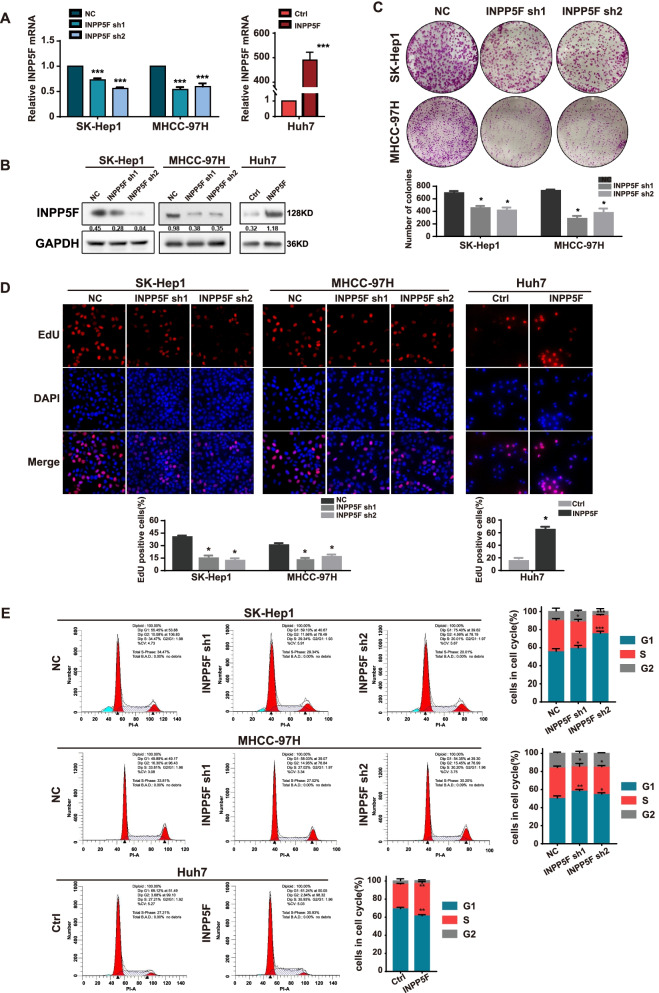
INPP5F promotes HCC cell proliferation in vitro. **A** QRT-PCR and (B) western blot confirmed the efficiencies of INPP5F knockdown in SK-Hep1 as well as MHCC-97H and overexpression in Huh7, respectively. **C** The effect of INPP5F on cell proliferation was determined by EdU assays. The representative images and the percentage of EdU positive cells are shown. **D** Colony formation was performed to validate the impact of INPP5F on cell proliferation. **E** The cell cycle analysis was performed in cells with INPP5F knockdown or overexpression. The percentage of cells in G1, S and G2 phase was indicated. Data is presented as means ± standard error for three independent experiments, *P < 0.05, ***P < 0.001

The biological function of INPP5F in HCC was next investigated. Because our above results showed significantly higher INPP5F expression in large HCC tumor cases, we focused on the association between INPP5F and the proliferation ability of HCC cell. INPP5F expression was knocked down in SK-Hep1 and MHCC-97H cells, and overexpressed in Huh7 cells (Fig. 2A and B, Fig. S2A-D). INPP5F knockdown reduced the EdU positive cells, while overexpression of INPP5F led to an increased effect (Fig. 2C, Fig. S2E). Consistent results were observed from colony formation assays (Fig. 2D). Moreover, INPP5F downregulation arrested HCC cell at G1 phase, while INPP5F overexpression reduced G1 phase (Fig. 2E, Fig. S2F). These findings indicate that INPP5F is a proliferation-promoting factor in HCC and this effect may through affecting G1/S phase transition.

**Fig. 3:**
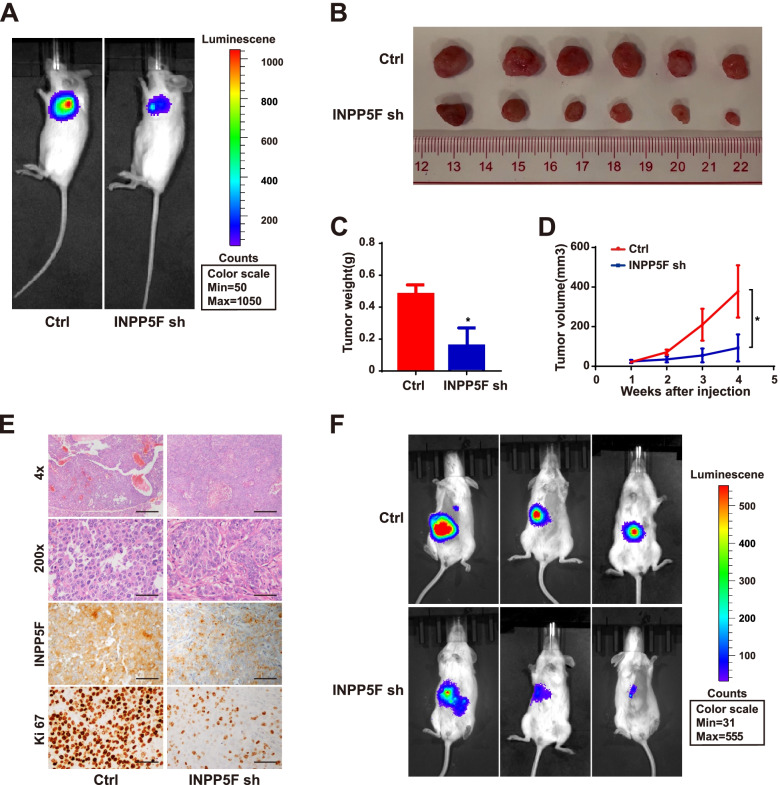
INPP5F facilitates HCC tumor growth in vivo. Six nude mice were used in subcutaneous xenograft model per group. **A** Representative images of subcutaneous xenograft model using INPP5F stable knockdown MHCC-97H and its control cells. **B** Tumors derived from the subcutaneous xenograft model in both groups. **C** The tumor weight in both groups was compared. **D** The tumor volumes were measured every week and indicated by curves. **E** H&E, INPP5F and Ki67 staining were conducted in serial sections of tumors from both groups. **F** Three NOD/SCID mice were used in orthotopic xenograft model per group to further evaluate the effect of INPP5F on cell growth in vivo. All *P < 0.05. Scale bar: 100 um

To confirm the above in vitro findings, we first employed subcutaneous xenograft model using MHCC-97H cells with stable INPP5F knockdown (Fig. 3A). The volume and weight of tumors formed in INPP5F knockdown group were significantly decreased compared with those formed in control group (Fig. 3B-D). H&E and IHC staining showed less necrosis and Ki67 expression in INPP5F knockdown group (Fig. 3E). We then established orthotopic tumor models by orthotopically injecting indicated cells under the liver capsular of mice. Consistently, INPP5F downregulation effectively inhibited the growth of orthotopic xenograft tumors in mice livers (Fig. 3F). Together, these results suggest that INPP5F exerted its oncogenic role probably by promoting proliferation of HCC cell.

### INPP5F upregulates the expression of c-MYC and cyclin E1 in HCC

**Fig. 4:**
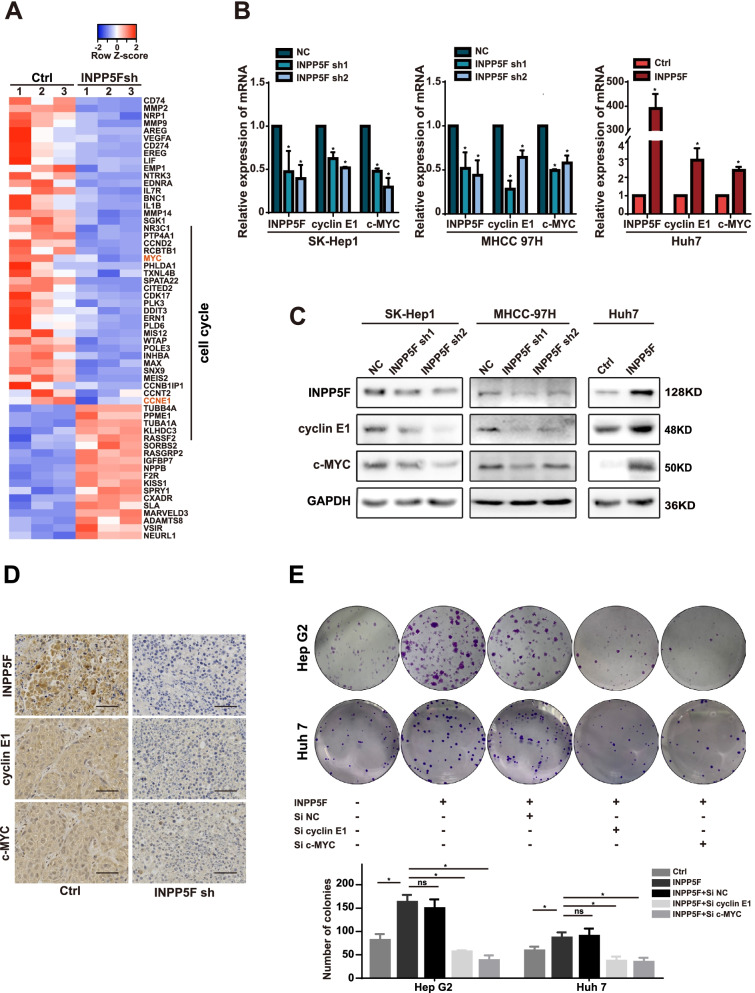
INPP5F upregulates the expression of c-MYC and cyclin E1 in HCC. **A** Heat map of DEGs associated with cell growth. The DEGs were obtained from SK-Hep1-Ctrl and SK-Hep1-shINPP5F through RNA-seq. **B** The mRNA and (**C**) protein expression of c-MYC and cyclin E1 in HCC cell lines with INPP5F knockdown or overexpression. **D** INPP5F, c-MYC and cyclin E1 staining were conducted in serial sections of tumors from subcutaneous xenograft model using INPP5F stable knockdown MHCC-97H and its control cells. **E** The impact of c-MYC and cyclin E1 knockdown on INPP5F-mediated cell proliferation was determined by colony formation assays. Data is presented as means ± standard error for three independent experiments, *P < 0.05, ns: not significant. Scale bar: 100 um

Given the findings above, we sought to explore the molecular mechanism underlying INPP5F-mediated tumor growth. Firstly, we compared whole-genome transcriptome between SK-Hep1-Ctrl and SK-Hep1-shINPP5F through RNA-seq, and obtained Differentially Expressed Genes (DEGs) via statistical analysis. By using 1.5-fold change as the cut-off point, 200 upregulated DEGs and 218 downregulated DEGs (Fig. S3A and B) were found. Gene Ontology (GO) analysis showed that there were 59 DEGs related to cell growth, including 29 genes associated with cell cycle (Fig. 4A). Since the above findings suggested that INPP5F may affect cell proliferation by regulating G1/S phase transition, we focused on the G1/S-related genes. Among the G1/S-related gene list, c-MYC and cyclin E1 are well-known genes tightly correlated with G1/S phase transition [15, 16]. Accordingly, INPP5F knockdown significantly decreased the mRNA expression of c-MYC and cyclin E1 in SK-Hep1 and MHCC-97H, whereas overexpression of INPP5F increased both genes expression in Huh7 cells (Fig. 4B). Results from western blot further supported the regulation of c-MYC and cyclin E1 by INPP5F (Fig. 4C). IHC results in subcutaneous tumor tissues also showed less cyclin E1 and c-MYC expression in INPP5F knockdown group (Fig. 4D). Furthermore, knockdown of c-MYC and cyclin E1 significantly inhibited the INPP5F-enhanced cell proliferation (Fig. 4E). Thus, our data suggest that INPP5F regulates HCC cell G1/S phase transition and proliferation through c-MYC and cyclin E1.

### INPP5F enhances aerobic glycolysis of HCC cell

Since previous studies have confirmed that aerobic glycolysis is closely related to cell proliferation [17], we further explored whether INPP5F affects aerobic glycolysis. By analyzing the above DEGs lists obtained from RNA-seq, 18 DEGs related to glycolysis were found (Fig. S3C). We selected hexokinase 2 (HK2), Hypoxia-inducible factor 1-alpha (HIF1A), glycolytic enzymes glucose transporter 1 (GLUT1) as well as GLUT3 that have been fully confirmed to participate in aerobic glycolysis in tumor for further validation. INPP5F knockdown decreased the expression of HK2 and HIF1A (Fig. S3D). In contrast, overexpression of INPP5F upregulated HK2 and HIF1A expression (Fig. S3D). We speculated that INPP5F is associated with aerobic glycolysis in HCC. Indeed, we observed the cell culture media of INPP5F knockdown cells was redder than the control cells (Fig. S3E), suggesting there may be lower acid concentration in the media of INPP5F knockdown cells. We thus further detected the lactate production and glucose consumption in INPP5F overexpression and knockdown cells. The lactate production and glucose consumption were reduced after INPP5F was knocked down, but increased when INPP5F was overexpressed (Fig. S3F and G). Collectively, these preliminary data suggest that INPP5F enhanced aerobic glycolysis of HCC cell.

### INPP5F activates notch signaling pathway via interacting with ASPH

**Fig. 5:**
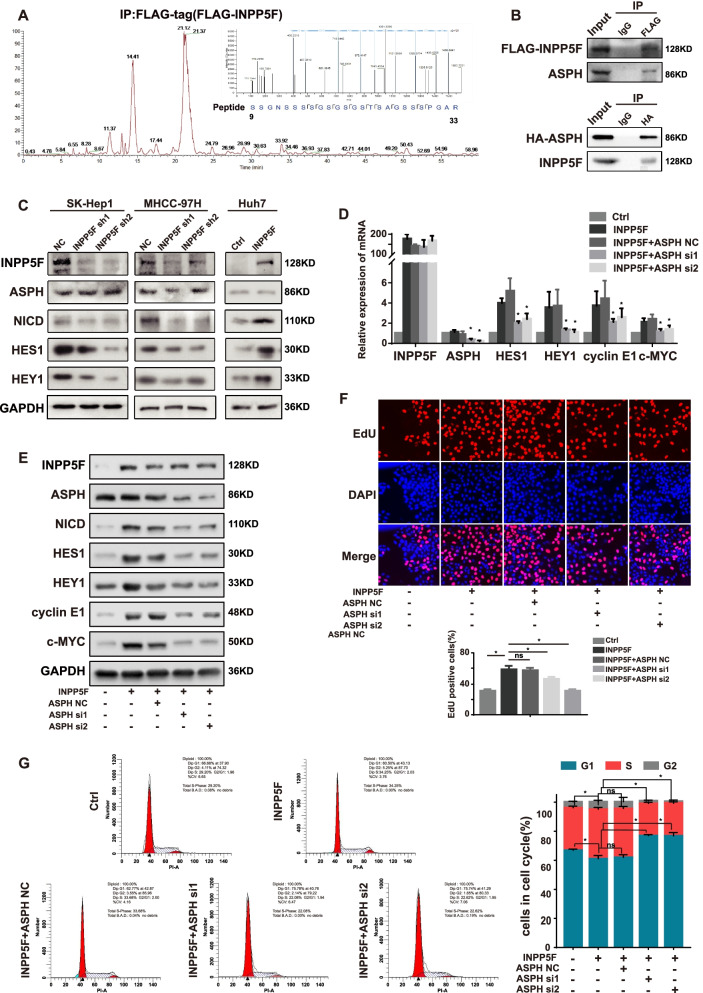
INPP5F activates Notch signaling pathway through interacting with ASPH. **A** The base-peak plot of mass spectrometry analysis of protein complex immunoprecipitated by anti-FLAG antibody in Huh7 overexpressing FLAG-INPP5F. The INPP5F pulled-down peptide of ASPH was indicated. **B** Lysates of Huh7 transiently overexpressing FLAG-INPP5F or HA-ASPH were immunoprecipitated for FLAG or HA and immunoblotted for ASPH or INPP5F, respectively. **C** Western blot was performed to investigate the influence of INPP5F on the expression of ASPH, NICD, HES1 and HEY1 in SK-Hep1, MHCC-97H and Huh7 cells. (D-G) Huh7 overexpressing INPP5F were transfected with ASPH siRNA for 24 h, and then were subjected to (**D**) QRT-PCR, (**E**) western blot, (**F**) EdU assay, and (**G**) cell cycle analysis. Knockdown of ASPH significantly attenuated INPP5F-enhanced expression of HES1, HEY1, cyclin E1 and c-MYC, as well as cell proliferation and G1/S phase transition. Data is presented as means ± standard error for three independent experiments, *P < 0.05, ns: not significant

We then decided to identify how INPP5F regulates the expression of its downstream molecules in HCC. Results from immunofluorescence indicated that INPP5F was mainly located in cytoplasm of HCC cell (Fig. S4A). Cytoplasmic signaling such as AKT-mTOR and STAT3 pathway have been reported to be downstream of INPP5F [18]. However, we found that INPP5F did not regulate the activation of AKT-mTOR and STAT3 signaling in HCC cell (Fig. S4B). We therefore employed immunoprecipitation combined with mass spectrometry to explore interacting proteins potentially mediating the biological function of INPP5F in HCC. Results showed that aspartate-β-hydroxylase (ASPH), a protein frequently upregulated in HCC [19], was a potential interacting partner of INPP5F (Fig. 5A). The interaction between INPP5F and ASPH was confirmed by co-immunoprecipitation (Fig. 5B). Further study showed that INPP5F did not alter the expression of ASPH at both mRNA and protein levels (Fig. S4C and D), suggesting INPP5F may affect the function of ASPH. Previous reports showed that the Notch pathway is a downstream signaling of ASPH [20, 21]. Coincidentally, c-MYC and cyclin E1 are important targets of the Notch signaling pathway [22, 23]. Thus, the effect of INPP5F on the activation of Notch signaling was determined. Western blot showed that the expression of Notch 1 intracellular domain (NICD) as well as its downstream HES1 and HEY1 was decreased in HCC cell with INPP5F knockdown, whereas increased in cells with INPP5F overexpression (Fig. 5C). Moreover, upon treatment of ASPH siRNA, INPP5F-mediated Notch pathway activation and c-MYC and cyclin E1 upregulation were dramatically suppressed (Fig. 5D and E). Functionally, knockdown of ASPH significantly attenuated INPP5F-enhanced cell proliferation, G1/S phase transition (Fig. 5F and G) and aerobic glycolysis (Fig. S4E and F). Together, these data suggest that INPP5F activates Notch signaling pathway in HCC via interacting with ASPH, leading to cell proliferation and aerobic glycolysis.

### INPP5F translocates into cytoplasm to exhibit its oncogenic activity

**Fig. 6:**
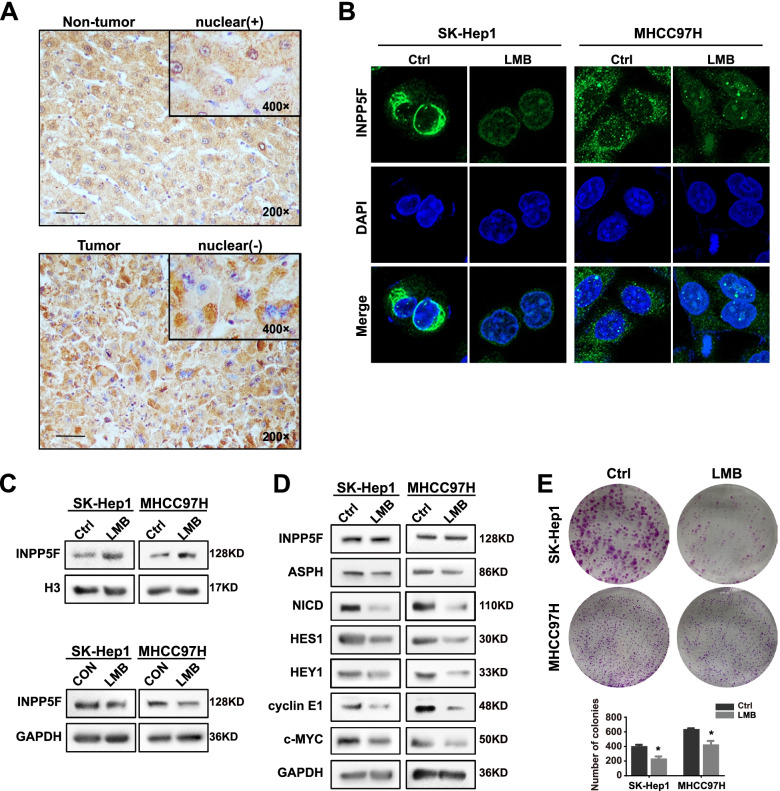
INPP5F translocates into cytoplasm to exhibit its oncogenic activity. **A** INPP5F was commonly nuclear staining in cells of adjacent non-tumor tissues, and cytoplasmic staining in HCC cells. **B**-**D** Indicated cells were treated with LMB (SK-Hep1: 10 ng/ml and MHCC 97H: 40 ng/ml) for 6 h, and then were subjected to (**B**) immunofluorescence for detection of sub-cellular localization of INPP5F, (**C**) western blot for detection of INPP5F protein expression in nuclear and cytoplasm. **D** western blot for detection of the expression of INPP5F-related downstream molecules. **E** LMB-induced nuclear restriction of INPP5F affected cell proliferation. Scale bar: 100 um

When evaluating the IHC staining of INPP5F, we surprisingly found that INPP5F was commonly nuclear-located in cells of adjacent non-tumor tissues, while in tumor tissues, cytoplasmic staining was more common (Fig. 6A and S5A). In addition, nuclear-positive staining of INPP5F in adjacent non-tumor tissues was associated with better prognosis in HCC patients (Fig. S5B). The different sub-cellular localization of INPP5F between tumor and adjacent non-tumor tissues in HCC prompted us to hypothesize that cytoplasmic translocation is important for INPP5F to display its oncogenic function. We used leptomycin B (LMB) to inhibit nuclear export. Both immunofluorescence and western blot indicated that INPP5F was restricted in nucleus after LMB treatment (Fig. 6B and C). Moreover, the nuclear restriction of INPP5F by LMB was associated with inhibition of Notch signaling and cell proliferation (Fig. 6D and E). These results suggested that INPP5F functions in HCC through a cytoplasmic translocation mechanism.

**Fig. 7:**
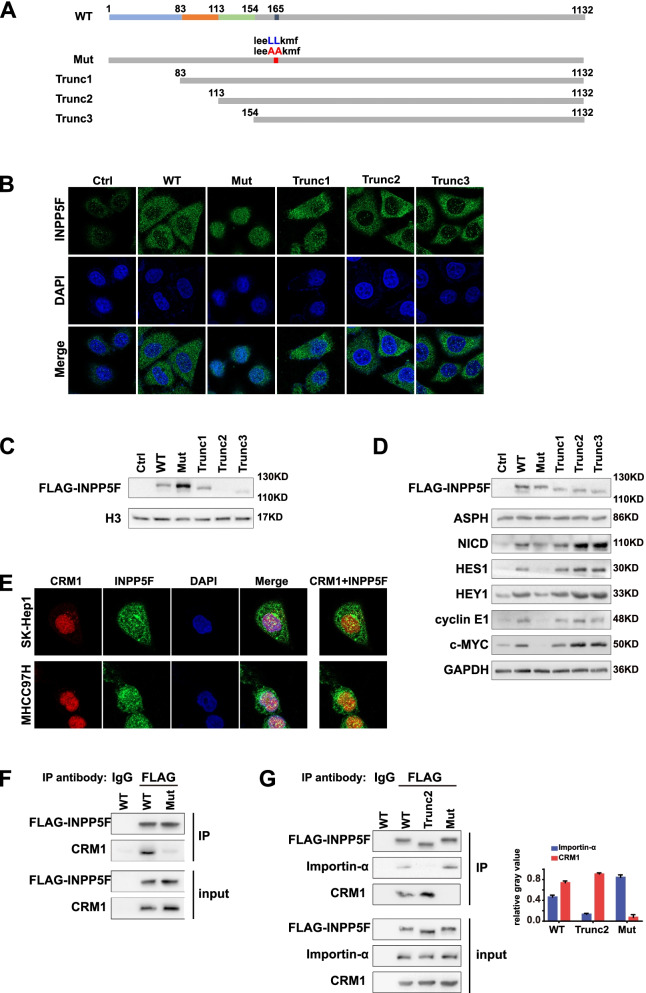
A competitive relationship between NESs mediated INPP5F-CRM1 interaction and INPP5F-importin-α interaction. **A** Schema of the NESs mutant and the three truncations (Trunc1, Trunc2 and Trunc3) of INPP5F. The prediction of NLSs was obtained from http://nls-mapper.iab.keio.ac.jp/cgi-bin/NLS_Mapper_form.cgi, and the prediction of NESs was obtained from http://www.cbs.dtu.dk/services/NetNES/ . **B**-**D** Cells were transfected with wild type (WT), NESs mutant or the three truncations. (B) Sub-cellular localization of different INPP5F was detected by immunofluorescence. **C** The nuclear localization of different INPP5F was validated by western blot. **D** The expression of INPP5F-related downstream molecules was detected by western blot. **E** Immunofluorescent staining displayed CRM1 and INPP5F mainly co-localized in the cell nucleus. **F** Huh7 Cells were transfected with Flag-tagged INPP5F WT and NESs mutant. Cell lysates were immunoprecipitated with anti-Flag antibody and immunoblotted with anti-CRM1 antibody. **G** Huh7 Cells were transfected with Flag-tagged INPP5F WT, NLSs Trunc2 and NESs mutant. Cell lysates were immunoprecipitated with anti-Flag antibody and immunoblotted with anti-Importin-α as well as anti-CRM1 antibody

Nuclear localization signals (NLSs) and nuclear export signals (NESs) are indispensable for protein’s translocation [24]. We thus employed bioinformatic tools to predict NLSs and NESs in INPP5F aminoacidic sequence. According to the prediction, one mutant with the potential NESs mutation (retained the NLSs) and three truncations with the potential NLSs deletion (retained the NESs) were constructed (Fig. 7A). Immunofluorescence and western blot showed that the NESs mutant restricted INPP5F in nucleus, while truncation 2 and 3 but not truncation 1 limited INPP5F in cytoplasm (Fig. 7B and C), suggesting the NESs sequence is responsible for the nuclear export of INPP5F, and NLSs, probably located in 83 to 113 of the INPP5F aminoacidic sequence, is related to the nuclear import of INPP5F. We found that compared with the NESs mutant, all the truncations significantly upregulated the colony formation, lactate production and glucose consumption of HCC cell, especially truncation 2 and 3 (Fig. S6A-C). Furthermore, results from both western blot (Fig. 7D) and QRT-PCR (Fig. S6D) showed that the expression of NICD, HES1, HEY1, c-MYC and cyclin E1 was significantly upregulated by truncation 2 and 3, while NESs mutant downpregulated the expression of these molecules.

LMB is a nuclear export inhibitor by targeting exportin CRM1. Considering the effect of LMB on the localization and function of INPP5F, we further explored whether INPP5F is transported into cytoplasm through CRM1. Immunofluorescence showed that INPP5F and CRM1 mainly colocalized in the cell nucleus (Fig. 7E). Co-immunoprecipitation validated the direct interaction between INPP5F and CRM1 (Fig. 7F). Upon NESs mutation, the interaction between INPP5F and CRM1 was significantly decreased (Fig. 7F), indicating that CRM1 mediates nuclear export of INPP5F by recognizing the NESs. Further co-immunoprecipitation data showed that both wild type INPP5F and NESs mutant could interact with importin-α, while truncation 2 could not (Fig. 7G), indicating that importin also regulates the cellular localization of INPP5F through NLSs. The data also showed that compared with wild type INPP5F, truncation 2 interacted with CRM1 more effectively, while NESs mutant preferred to binding with importin-α (Fig. 7G). Moreover, the interaction of wild type INPP5F with CRM1 was stronger than with importin-α (Fig. 7G). These results implied that CRM1 and importin-α may competitively interact with INPP5F, but in HCC cell the INPP5F-CRM1 binding may be more dominant. Taken together, our data suggested that the preferred interaction of INPP5F with CRM1 results in its cytoplasmic translocation in HCC cell, where INPP5F exerts its oncogenic function.

## Discussion

**Fig. 8:**
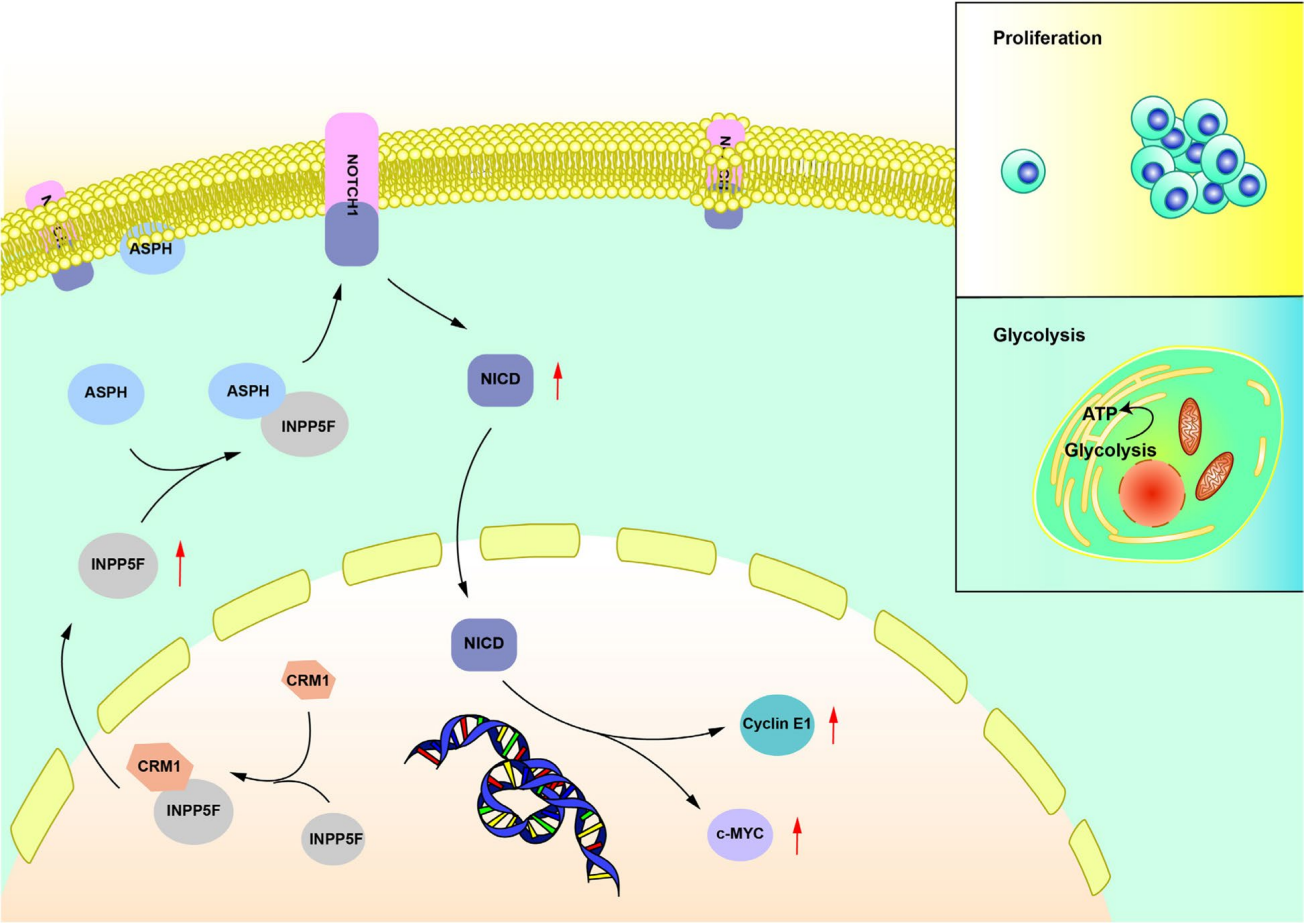
Model for the mechanism of INPP5F in facilitating HCC tumor growth. INPP5F translocates into cytoplasm by binding with CRM1, where INPP5F interacts with ASPH and activates Notch-c-MYC/cyclin E1 pathway, resulting in enhancement of HCC cell aerobic glycolysis and proliferation

Deregulation of phosphatases contributes to tumorigenesis and tumor progression, so phosphatases are exciting targets for HCC drug discovery [25]. As for the role of phosphatase in HCC, our previous studies have reported that PRL-1 promotes HCC cell migration and invasion through endothelial-mesenchymal transition induction [26], and that PRL-3 facilitates HCC progression by co-amplifing with FAK as well as enhancing FAK phosphorylation [27]. Inositol polyphosphate 5-phosphatases are a group of phosphatases involved in regulating phosphorylation of phosphoinositide. Increasing evidence has suggested inositol polyphosphate 5-phosphatases contribute to tumorigenesis and tumor progression. For instance, depletion of INPP5J reduces cell migration and invasion by regulating AKT1 signaling in breast cancer [5]. Loss of INPP5A expression predicts poor overall survival in recurrent and metastatic disease of cutaneous squamous cell carcinoma [6]. INPP5E is reported to promote Sonic Hedgehog medulloblastoma progression via a phosphoinositide signaling axis at cilia [7]. In this study, we focused on INPP5F. We found that INPP5F is overexpressed in HCC tissues. High expression of INPP5F predicts poor prognosis in patients with HCC. Mechanically, we identified ASPH is an interacting protein of INPP5F. INPP5F promotes HCC cell proliferation, aerobic glycolysis and activating Notch-c-MYC/cyclin E1 pathway through ASPH. Furthermore, we found that the oncogenic function of INPP5F in HCC is dependent on the CRM1-mediated cytoplasmic translocation. (Fig. 8). Thus, our data indicate that INPP5F is an oncogene in HCC. However, whether the oncogenic mechanism of INPP5F in HCC is link to its inositol-phosphatase activity still needs further investigation in future.

ASPH is a member of the α-ketoglutarate-dependent dioxygenase family. It can catalyze the hydroxylation of aspartyl and asparaginyl residues in the EGF-like domains of various proteins, such as Notch1 and JAG2, leading to activation of Notch signaling pathway [28, 29]. Overexpression of ASPH has been reported in more than 20 tumor types [20]. Indeed, recent study has revealed that ASPH is highly expressed in HCC and is one of the major activators of Notch pathway, playing an important role in HCC progression [19, 30]. In this study, we identified INPP5F as an interactor of ASPH in HCC. INPP5F activates Notch pathway and enhances cell growth and aerobic glycolysis through ASPH. Besides, knockdown of INPP5F inhibited Notch signaling without affecting ASPH expression. These data suggest INPP5F may also be necessary for ASPH to activate Notch pathway. Our results not only explore the oncogenic role of INPP5F in HCC, but also improve our understanding of how ASPH functions in HCC.

Our data showed that INPP5F not only promotes cell proliferation, but also enhances aerobic glycolysis of HCC cell. Aerobic glycolysis, also known as the Warburg effect, is a general feature of glucose metabolism in cancer cells [31]. Unlike normal cells, cancer cells prefer glycolysis for ATP production, even under aerobic conditions [32]. Along with ATP production, aerobic glycolysis also generates various of metabolic intermediates which are essential for the rapid growth of tumor cells [33]. Thus, the enhancement of aerobic glycolysis by INPP5F could subsequently provide substrates for anabolic pathways and finally promotes cell proliferation. Furthermore, either Notch pathway or c-Myc alone have been reported to enhance the aerobic glycolysis of cancer cells [34, 35]. Hence, there may exist an INPP5F-dependent network connecting cell proliferation and aerobic glycolysis in HCC cell, leading to HCC progression.

Another interesting finding of our study is the diverse sub-cellular localization of INPP5F between adjacent non-tumor tissues and HCC tissues. Shuttling of specific proteins out of nucleus is essential for the regulation of intracellular signaling and can influence the biological function of tumor cells [36]. Actually, nuclear-cytoplasmic shuttling is a targetable process [37]. CRM1 is the main mediator of nuclear export in many cell types. Regulating the sub-cellular localization of NESs and NLSs-containing oncogenes is the major function of CRM1 in malignant tumors [38]. Recently, one of CRM1 inhibitors, selinexor, has been approved by FDA for multiple myeloma patients with a refractory disease [39]. In the current study, we noticed the nuclear-cytoplasmic shuttling of INPP5F could be impaired by LMB, a small molecule inhibitor of CRM1, accompanied by inhibition of Notch pathway and cell proliferation. We found a direct interaction between INPP5F and CRM1, resulting in cytoplasmic translocation of INPP5F. These data suggest CRM1 inhibitor may also perform its anti-tumor effect through regulating INPP5F in HCC. In addition, our data showed that NESs mutation affected nuclear export and led to nuclear accumulation of INPP5F as LMB treatment did. Meanwhile, NLSs deletion led to cytoplasmic localization of INPP5F, providing an opportunity to bind to ASPH and thereby activate the Notch pathway to exert its cancer-promoting function. Thus, mutating the NESs and/or retaining the NLSs of INPP5F may be another strategy for targeting INPP5F in HCC. Furthermore, we noted that CRM1 could transport INPP5F out of the nucleus by recognizing NESs, and importin-α could transport INPP5F into the nucleus by recognizing NLSs. Of these two processes, the effect of CRM1 may play a dominant role in HCC cells. We speculate that there may be molecules that prevent INPP5F from binding to importin and promote its binding to exportin, resulting in the cytoplasmic localization of INPP5F in HCC. Actually, abnormal expression of proteins and non-coding RNA in tumor have been demonstrated to regulate nuclear-cytoplasmic shuttling. For instance, Feng-Qian Li et al. found that protein 14–3-3 could regulate the nuclear-cytoplasmic shuttling of Cby. It enhances the Cby-CRM1 interaction while interfering with the Cby-importin-α interaction, thereby achieving the cytoplasmic localization of Cby [40]. Sanhita Mitra et al. found that the reduction of lncRNA NBAT1 could accumulate p53 in the cytoplasm by enhancing the function of CRM1, resulting in drug resistance of neuroblastoma cells [41]. Future mechanistic studies may focus on discovering the molecule that regulate the competitive binding of CRM1 and importin-α to INPP5F, making it a more attractive target for anti-tumor therapies.

## Conclusions

In summary, the present study identifies a novel oncogenic role of INPP5F in HCC. The upregulation of INPP5F predicts poor prognosis in HCC patients. Mechanically, we found that INPP5F translocates into cytoplasm by binding with CRM1 where INPP5F interacts with ASPH and activates Notch signaling, resulting in enhancement of HCC cell aerobic glycolysis and proliferation. Thus, our data indicated that INPP5F, as a newly identified nuclear-cytoplasmic shuttling protein, may be a potential therapeutic target for HCC.

## Supplementary Information

**Additional file 1.**

## Data Availability

The datasets used and/or analyzed during the current study are available from the corresponding author on reasonable request.

## Abbreviations

INPP5F: Inositol polyphosphate-5-phosphatase F
ASPH: Aspartate-β-hydroxylase
GAPDH: Glyceraldehyde-3-phosphate dehydrogenase
HE: Hematoxylin-eosin
DAB: Diaminobenzidine
DMEM: Dulbecco Modified Eagle Medium
PVDF: Polyvinylidene fluoride
PBS: Phosphate buffer saline
BSA: Bovine serum albumin
NOD/SCID: Non-obese diabetic/severe combined immune deficency
DAPI: 4′,6-diamidino-2-phenylindole

## References

[1] Sung H, Ferlay J, Siegel RL, Laversanne M, Soerjomataram I, Jemal A (2021). Global Cancer statistics 2020: GLOBOCAN estimates of incidence and mortality worldwide for 36 cancers in 185 countries. CA Cancer J Clin.

[2] Marrero JA, Kulik LM, Sirlin CB, Zhu AX, Finn RS, Abecassis MM (2018). Diagnosis, staging, and Management of Hepatocellular Carcinoma: 2018 practice guidance by the American Association for the Study of Liver Diseases. Hepatology.

[3] De Matteis MA, Staiano L, Emma F, Devuyst O (2017). The 5-phosphatase OCRL in Lowe syndrome and dent disease 2. Nat Rev Nephrol.

[4] Ramos AR, Ghosh S, Erneux C (2019). The impact of phosphoinositide 5-phosphatases on phosphoinositides in cell function and human disease. J Lipid Res.

[5] Ooms LM, Binge LC, Davies EM, Rahman P, Conway JR, Gurung R (2015). The inositol polyphosphate 5-phosphatase PIPP regulates AKT1-dependent breast Cancer growth and metastasis. Cancer Cell.

[6] Maly CJ, Cumsky HJL, Costello CM, Schmidt JE, Butterfield RJ, Zhang N (2020). Prognostic value of inositol polyphosphate-5-phosphatase expression in recurrent and metastatic cutaneous squamous cell carcinoma. J Am Acad Dermatol.

[7] Conduit SE, Ramaswamy V, Remke M, Watkins DN, Wainwright BJ, Taylor MD (2017). A compartmentalized phosphoinositide signaling axis at cilia is regulated by INPP5E to maintain cilia and promote sonic hedgehog medulloblastoma. Oncogene.

[8] Nakatsu F, Messa M, Nandez R, Czapla H, Zou Y, Strittmatter SM (2015). Sac2/INPP5F is an inositol 4-phosphatase that functions in the endocytic pathway. J Cell Biol.

[9] Zhu W, Trivedi CM, Zhou D, Yuan L, Lu MM, Epstein JA (2009). Inpp5f is a polyphosphoinositide phosphatase that regulates cardiac hypertrophic responsiveness. Circ Res.

[10] Cao M, Park D, Wu Y, De Camilli P (2020). Absence of Sac2/INPP5F enhances the phenotype of a Parkinson's disease mutation of synaptojanin 1. Proc Natl Acad Sci U S A.

[11] Kim HS, Li A, Ahn S, Song H, Zhang W (2014). Inositol Polyphosphate-5-phosphatase F (INPP5F) inhibits STAT3 activity and suppresses gliomas tumorigenicity. Sci Rep.

[12] Palermo G, Maisel D, Barrett M, Smith H, Duchateau-Nguyen G, Nguyen T (2015). Gene expression of INPP5F as an independent prognostic marker in fludarabine-based therapy of chronic lymphocytic leukemia. Blood Cancer J.

[13] Johnston HE, Carter MJ, Larrayoz M, Clarke J, Garbis SD, Oscier D (2018). Proteomics profiling of CLL versus healthy B-cells identifies putative therapeutic targets and a subtype-independent signature of spliceosome dysregulation. Mol Cell Proteomics.

[14] Zhou Z, Jiang H, Tu K, Yu W, Zhang J, Hu Z (2019). ANKHD1 is required for SMYD3 to promote tumor metastasis in hepatocellular carcinoma. J Exp Clin Cancer Res.

[15] Lu C, Xia J, Zhou Y, Lu X, Zhang L, Gou M (2016). Loss of Gsalpha impairs liver regeneration through a defect in the crosstalk between cAMP and growth factor signaling. J Hepatol.

[16] Icard P, Fournel L, Wu Z, Alifano M, Lincet H (2019). Interconnection between metabolism and cell cycle in Cancer. Trends Biochem Sci.

[17] Ganapathy-Kanniappan S (2018). Molecular intricacies of aerobic glycolysis in cancer: current insights into the classic metabolic phenotype. Crit Rev Biochem Mol Biol.

[18] Yuan L, Liu C, Wan Y, Yan H, Li T (2019). Effect of HDAC2/Inpp5f on neuropathic pain and cognitive function through regulating PI3K/Akt/GSK-3beta signal pathway in rats with neuropathic pain. Exp Ther Med.

[19] Aihara A, Huang CK, Olsen MJ, Lin Q, Chung W, Tang Q (2014). A cell-surface beta-hydroxylase is a biomarker and therapeutic target for hepatocellular carcinoma. Hepatology.

[20] Lin Q, Chen X, Meng F, Ogawa K, Li M, Song R (2019). ASPH-notch Axis guided Exosomal delivery of Prometastatic Secretome renders breast Cancer multi-organ metastasis. Mol Cancer.

[21] Kanwal M, Smahel M, Olsen M, Smahelova J, Tachezy R (2020). Aspartate beta-hydroxylase as a target for cancer therapy. J Exp Clin Cancer Res.

[22] Sun L, Wang Y, Cen J, Ma X, Cui L, Qiu Z (2019). Modelling liver cancer initiation with organoids derived from directly reprogrammed human hepatocytes. Nat Cell Biol.

[23] Cui D, Dai J, Keller JM, Mizokami A, Xia S, Keller ET (2015). Notch pathway inhibition using PF-03084014, a gamma-secretase inhibitor (GSI), enhances the antitumor effect of docetaxel in prostate Cancer. Clin Cancer Res.

[24] Su EC, Chang JM, Cheng CW, Sung TY, Hsu WL (2012). Prediction of nuclear proteins using nuclear translocation signals proposed by probabilistic latent semantic indexing. BMC Bioinformatics.

[25] Frankson R, Yu ZH, Bai Y, Li Q, Zhang RY, Zhang ZY (2017). Therapeutic targeting of oncogenic tyrosine phosphatases. Cancer Res.

[26] Jin S, Wang K, Xu K, Xu J, Sun J, Chu Z (2014). Oncogenic function and prognostic significance of protein tyrosine phosphatase PRL-1 in hepatocellular carcinoma. Oncotarget.

[27] Zhou Q, Zhou Q, Liu Q, He Z, Yan Y, Lin J (2020). PRL-3 facilitates hepatocellular carcinoma progression by co-amplifying with and activating FAK. Theranostics.

[28] Iwagami Y, Huang CK, Olsen MJ, Thomas JM, Jang G, Kim M (2016). Aspartate beta-hydroxylase modulates cellular senescence through glycogen synthase kinase 3beta in hepatocellular carcinoma. Hepatology.

[29] Zou Q, Hou Y, Wang H, Wang K, Xing X, Xia Y (2018). Hydroxylase activity of ASPH promotes hepatocellular carcinoma metastasis through epithelial-to-mesenchymal transition pathway. EBioMedicine.

[30] Shimoda M, Tomimaru Y, Charpentier KP, Safran H, Carlson RI, Wands J (2012). Tumor progression-related transmembrane protein aspartate-beta-hydroxylase is a target for immunotherapy of hepatocellular carcinoma. J Hepatol.

[31] DeBerardinis RJ, Chandel NS (2016). Fundamentals of cancer metabolism. Sci Adv.

[32] Kitamura K, Hatano E, Higashi T, Narita M, Seo S, Nakamoto Y (2011). Proliferative activity in hepatocellular carcinoma is closely correlated with glucose metabolism but not angiogenesis. J Hepatol.

[33] Flaveny CA, Griffett K, El-Gendy Bel D, Kazantzis M, Sengupta M, Amelio AL (2015). Broad anti-tumor activity of a small molecule that selectively targets the Warburg effect and lipogenesis. Cancer Cell.

[34] Jitschin R, Braun M, Qorraj M, Saul D, Le Blanc K, Zenz T (2015). Stromal cell-mediated glycolytic switch in CLL cells involves notch-c-Myc signaling. Blood.

[35] Fang Y, Shen ZY, Zhan YZ, Feng XC, Chen KL, Li YS (2019). CD36 inhibits beta-catenin/c-myc-mediated glycolysis through ubiquitination of GPC4 to repress colorectal tumorigenesis. Nat Commun.

[36] Tran EJ, King MC, Corbett AH (1843). Macromolecular transport between the nucleus and the cytoplasm: advances in mechanism and emerging links to disease. Biochim Biophys Acta.

[37] Conforti F, Wang Y, Rodriguez JA, Alberobello AT, Zhang YW, Giaccone G (2015). Molecular pathways: anticancer activity by inhibition of nucleocytoplasmic shuttling. Clin Cancer Res.

[38] Gravina GL, Senapedis W, McCauley D, Baloglu E, Shacham S, Festuccia C (2014). Nucleo-cytoplasmic transport as a therapeutic target of cancer. J Hematol Oncol.

[39] Chari A, Vogl DT, Gavriatopoulou M, Nooka AK, Yee AJ, Huff CA (2019). Oral Selinexor-dexamethasone for triple-class refractory multiple myeloma. N Engl J Med.

[40] Li FQ, Mofunanya A, Fischer V, Hall J, Takemaru K (2010). Nuclear-cytoplasmic shuttling of Chibby controls beta-catenin signaling. Mol Biol Cell.

[41] Mitra S, Muralidharan SV, Di Marco M, Juvvuna PK, Kosalai ST, Reischl S (2021). Subcellular distribution of p53 by the p53-responsive lncRNA NBAT1 determines chemotherapeutic response in neuroblastoma. Cancer Res.

