# Supplementary material for: INPP5F translocates into cytoplasm and interacts with ASPH to promote tumor growth in hepatocellular carcinoma
Source: J Exp Clin Cancer Res. 2022 Jan 7;41:13. doi: 10.1186/s13046-021-02216-x (PMC8740451; doi:10.1186/s13046-021-02216-x)
Supplement: Supplementary file 1 — Additional file 1. [file 13046_2021_2216_MOESM1_ESM.doc]

**Supplementary Tables**

**Table S1 Correlation of INPP5F expression and clinical features of HCC patients**

| Variable | INPP5F | | | |
| --- | --- | --- | --- | --- |
|  | All cases | Low expression | High expression | P value |
| Age（years） |  |  |  | 0.8981 |
| <50 | 102 | 47(46.08%） | 55(53.92%) |  |
| ≥50 | 130 | 61(46.92%) | 69(53.08%) |  |
| Gender |  |  |  | 0.5744 |
| Male | 211 | 97(45.97% | 114(54.03%) |  |
| Female | 21 | 11(52.38%) | 10(47.62%) |  |
| HBV infection |  |  |  | 0.8973 |
| Negative | 23 | 11(47.83%) | 12(52.17%) |  |
| Positive | 209 | 97(46.41%) | 112(53.59%) |  |
| AFP (ng/ml) |  |  |  | 0.7579 |
| < 20 | 97 | 44（45.36%）） | 53（54.64%）） |  |
| ≥20 | 135 | 64（47.41%）） | 71（52.59%）） |  |
| Cirrhosis |  |  |  | **0.0338** |
| No | 64 | 37(57.81%) | 27(42.19%) |  |
| Yes | 168 | 71(42.26%) | 97(57.74%) |  |
| Tumor size (cm) |  |  |  | **0.0400** |
| < 5 | 110 | 59(53.64%) | 51(46.36%) |  |
| ≥5 | 122 | 49(40.16%) | 73(59.84%) |  |
| Tumor multiplicity |  |  |  | 0.8881 |
| Single | 173 | 81(46.82%) | 92(53.18%) |  |
| Multiple | 59 | 27(45.76%) | 32(54.24%) |  |
| Tumor differentiation |  |  |  | **0.0414** |
| Well-Moderate | 145 | 75(51.72%) | 70(48.28%) |  |
| Poor-undifferentiated | 87 | 33(37.93%) | 54(62.07%) |  |
| Vascular invasion |  |  |  | 0.2396 |
| No | 91 | 38(41.76%) | 53(58.24%) |  |
| Yes | 141 | 70(49.65%) | 71(50.35%) |  |
| LNM |  |  |  | 0.3458 |
| No | 220 | 104(47.27%) | 116(52.73%) |  |
| Yes | 12 | 4(33.33%) | 8(66.67) |  |
| Tumor encapsulation |  |  |  | 0.8767 |
| Complete | 104 | 49(47.12%) | 55(52.88%) |  |
| Incomplete | 128 | 59(46.09%) | 69(53.91%) |  |
| TNM |  |  |  | 0.4478 |
| I-II | 135 | 60(44.44%) | 75(55.56%) |  |
| III-IV | 97 | 48(49.48%) | 49(50.52%) |  |
| Ascites |  |  |  | 0.9358 |
| No | 195 | 91(46.67%) | 104(53.33%) |  |
| Yes | 17 | 17(45.95%) | 20(54.05%) |  |
| Portal hypertension |  |  |  | 0.7321 |
| No | 190 | 89(46.84%) | 101(53.16%) |  |
| Yes | 41 | 18(43.90%) | 23(56.10%) |  |

HBV hepatitis B virus, AFP alpha-fetoprotein, LNM lymph node metastasis, TNM tumor-node-metastasis.

p values were calculated by comparing the expression of INPP5F with different clinical variables respectively using a chi-square test. p < 0.05 was considered statistically significant.

**Table S2 Univariate and multivariate analyses of INPP5F expression and overall survival**

| Variables | Univariate analysis |  | Multivariate analysis |  |
| --- | --- | --- | --- | --- |
|  | HR (95% CI) | P value | HR (95% CI) | P value |
| Overall survival |  |  |  |  |
| Age  (<50 vs.≥50 years) | 0.946 (0.670-1.336) | 0.754 |  |  |
| Gender  (male vs.female) | 1.196 (0.674-2.122) | 0.540 |  |  |
| HBV infection  (negative vs. positive) | 0.816 (0.477-1.398) | 0.460 |  |  |
| Cirrhosis  (no vs. yes) | 1.091(0.744-1.601) | 0.656 |  |  |
| AFP (ng/ml)  (< 20 vs. ≥20 ng/mL) | 3.906 (2.605-5.857) | **<0.001** | 3.107 (1.981-4.872) | **<0.001** |
| Tumor size (cm)  (< 5 vs. ≥5 cm) | 2.494 (1.743-3.569) | **<0.001** | 1.211 (0.793-1.849) | 0.375 |
| Tumor multiplicity  (single vs. multiple) | 1.613 (1.111-2.342) | **0.012** | 1.208 (0.807-1.808) | 0.358 |
| Tumor differentiation | 1.701 (1.204-2.404) | **0.003** | 1.295 (0.892-1.880) | 0.174 |
| Vascular invasion  (no vs. yes) | 2.155 (1.486-3.124) | **<0.001** | 1.170 (0.772-1.774) | 0.458 |
| LNM  (no vs. yes) | 4.140 (2.141-8.003) | **<0.001** | 1.323 (0.631-2.776) | 0.459 |
| Tumor encapsulation  (complete vs. incomplete) | 1.646 (1.159-2.338) | **0.005** | 1.183 (0.796-1.757) | 0.406 |
| TNM  (I-II vs. III-IV) | 3.606 (2.535-5.128) | **<0.001** | 2.352 (1.517-3.646) | **<0.001** |
| Ascites  (no vs. yes) | 1.842 (1.196-2.837) | **0.006** | 1.268 (0.772-2.083) | 0.348 |
| Portal hypertension  (no vs. yes) | 1.637 (1.076-2.491) | **0.021** | 1.020 (1.127-2.463) | 0.934 |
| INPP5F  (low vs. high) | 1.459 (1.025-2.077) | **0.036** | 1.666 (1.127-2.463) | **0.010** |

HBV hepatitis B virus, AFP alpha-fetoprotein, LNM lymph node metastasis, TNM tumor-node-metastasis, HR hazard ratio, CI confidence interval.

**Supplementary Figures
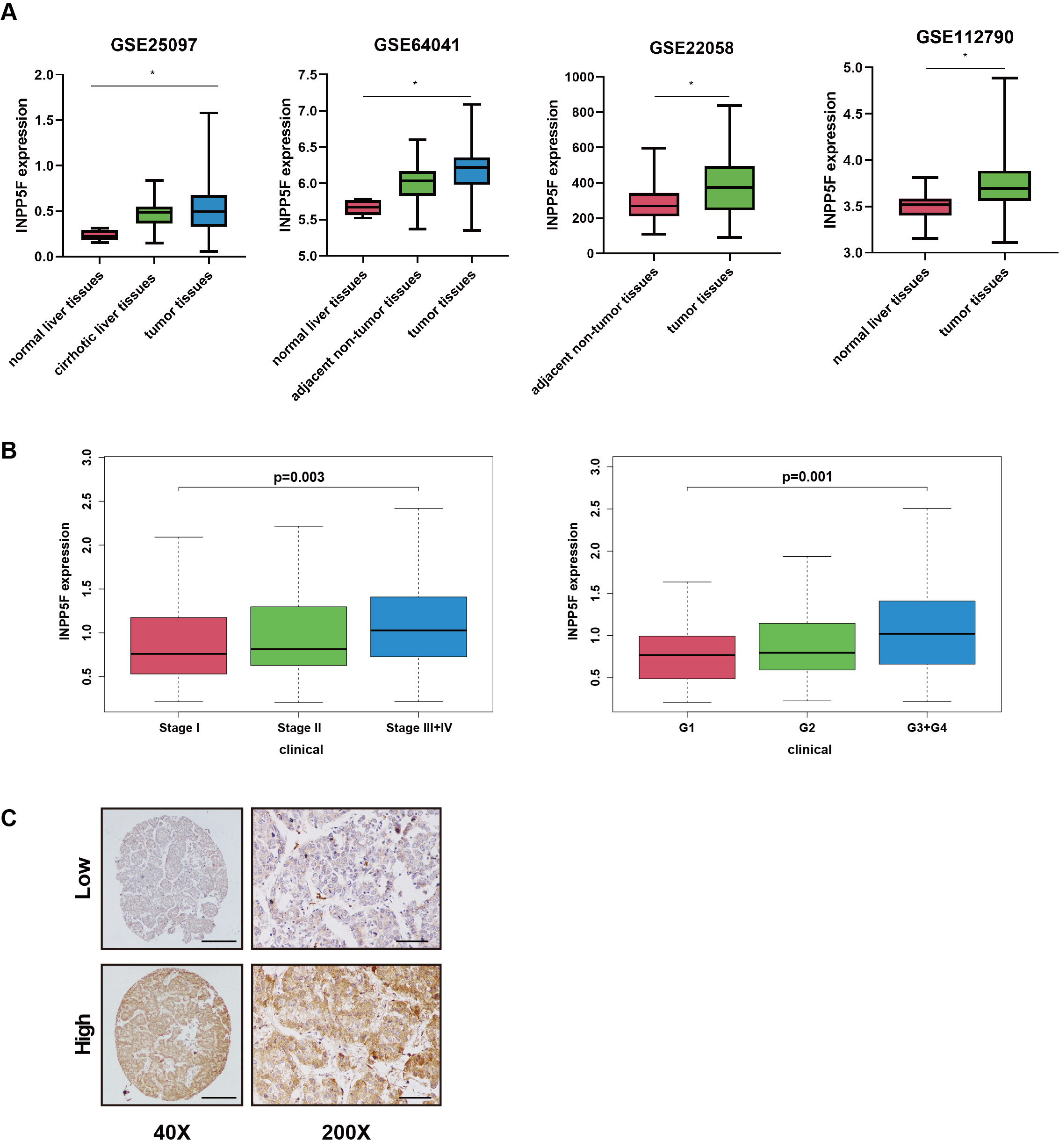
**

**Figure. S1 Expression level and prognosis value of INPP5F in HCC**. (A) HCC datasets from GEO database ([http://www.ncbi.nlm.nih.gov/geo](https://www.ncbi.nlm.nih.gov/geo/)) showed increase of INPP5F mRNA in HCC samples. (B) Bioinformatic analysis and comparison of mRNA expression level of INPP5F in HCC patients grouped by tumor stage and grade in TCGA-LIHC cohort. Data were obtained from UALCAN cancer database (<http://ualcan.path.uab.edu/>). (C) Representative images of IHC staining with Low-PRL-3 expression and high-PRL-3 expression. **P* < 0.05. Scale bar: 100 um.

**
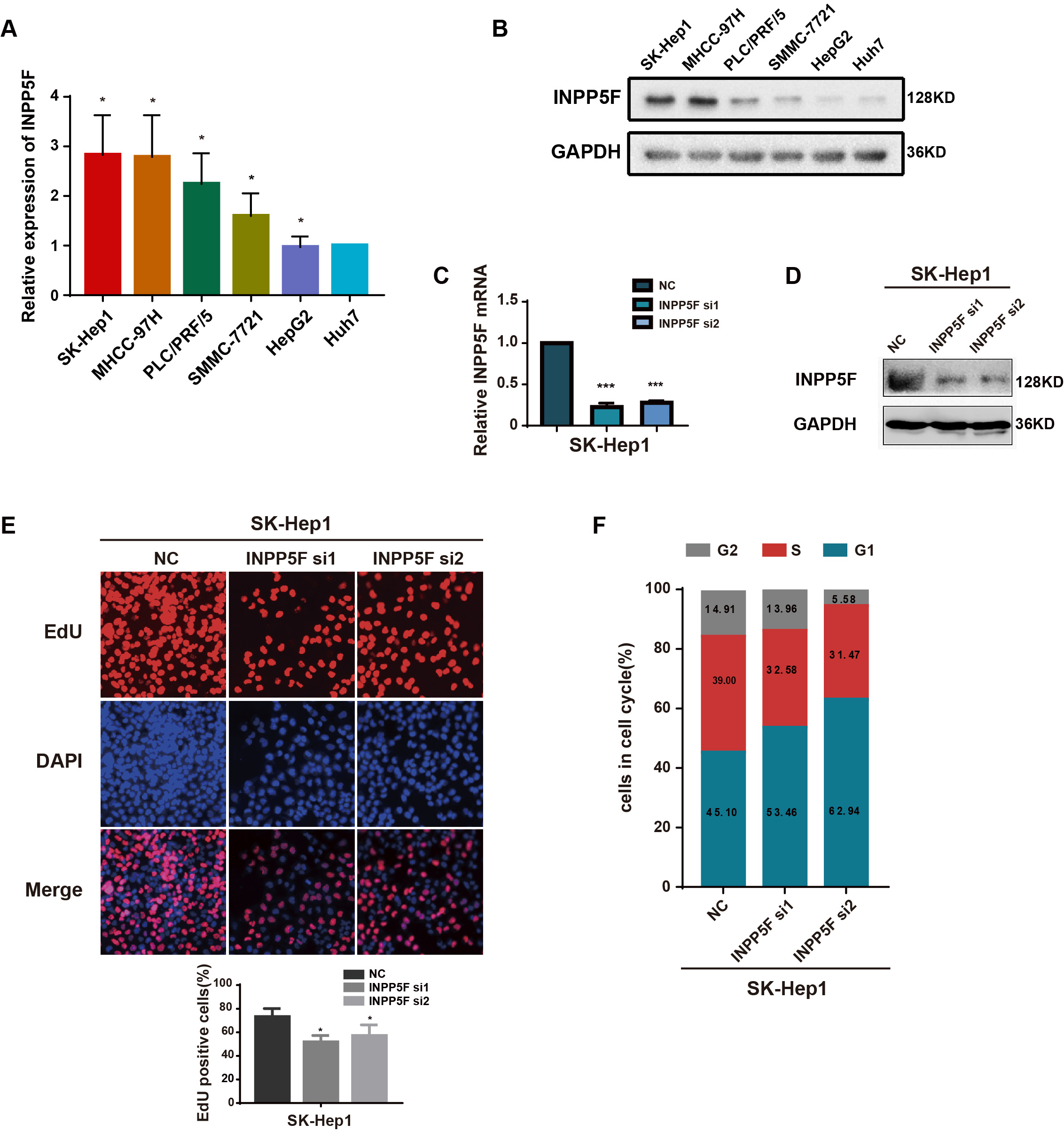
**

**Figure. S2 The effect of INPP5F on HCC cell proliferation and migration.** (A) QRT-PCR and (B) Western blot compared INPP5F expression levels in different HCC cell lines. (C) QRT-PCR and (D) western blot confirmed the efficiencies of INPP5F knockdown using siRNAs in SK-Hep1. (E) The effect of silenced INPP5F on cell proliferation was determined by EdU assays. (F) The cell cycle analyses were performed, and the percentage of cells in G1, S and G2 phase was indicated. Data is presented as means ± standard error for three independent experiments, *P < 0.05, ***P < 0.001. Scale bar: 100 um.

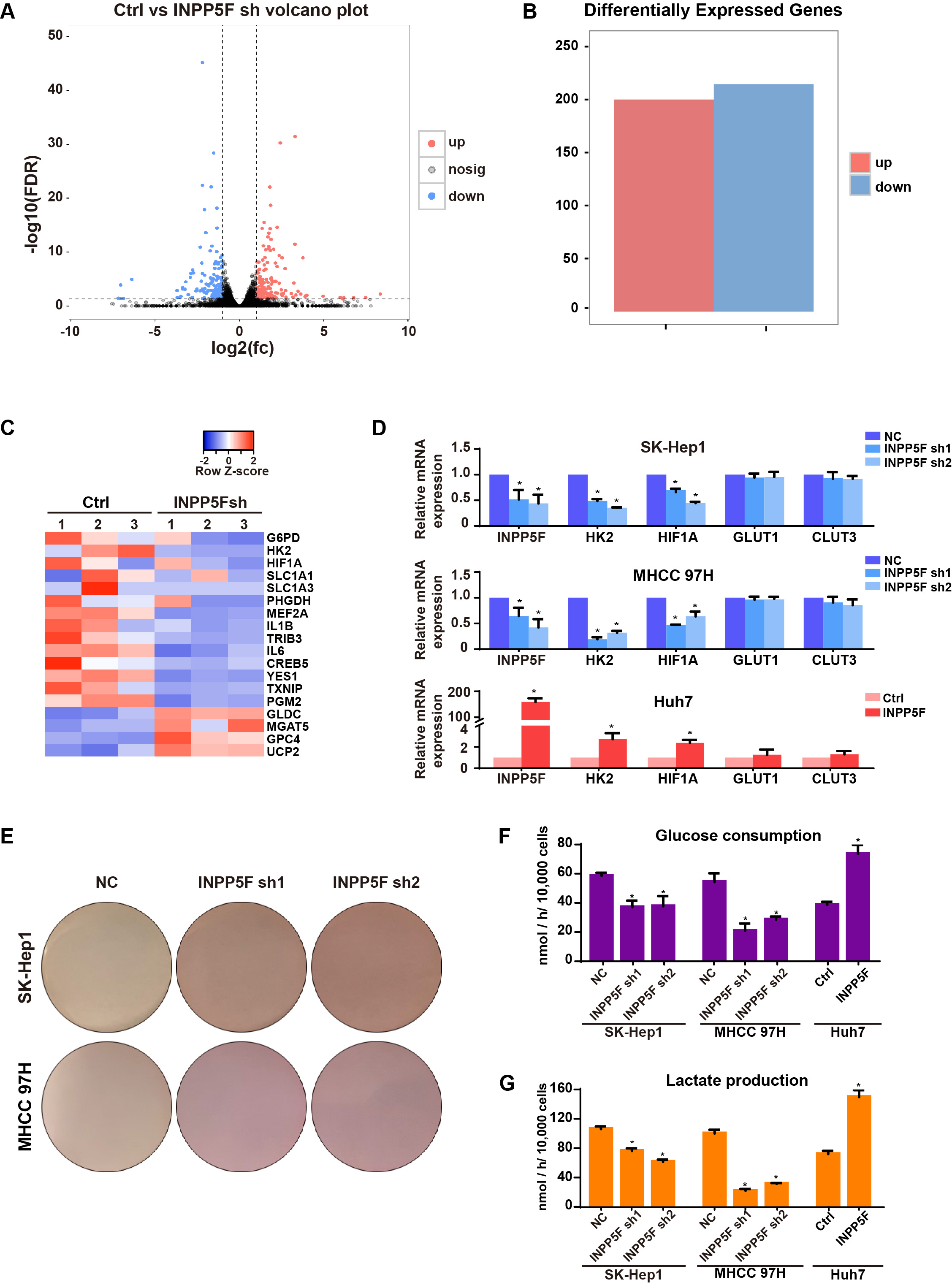


**Figure. S3 INPP5F enhances aerobic glycolysis of HCC cells.** (A, B) The volcano plot and number of DEGs. (C) Heat map of DEGs associated with aerobic glycolysis. The DEGs were obtained from SK-Hep1-Ctrl and SK-Hep1-shINPP5F through RNA-seq. (D)The mRNA expression of HK2, HIF1A, GLUT1 and GLUT3 in INPP5F knockdown or overexpressing cells. (E) Representative images of cell culture media color in INPP5F stable knockdown SK-Hep1 and MHCC-97H cells. (F, G) Glucose consumption and lactate production of cells with INPP5F knockdown or overexpression. Data is presented as means ± standard error for three independent experiments, *P < 0.05.


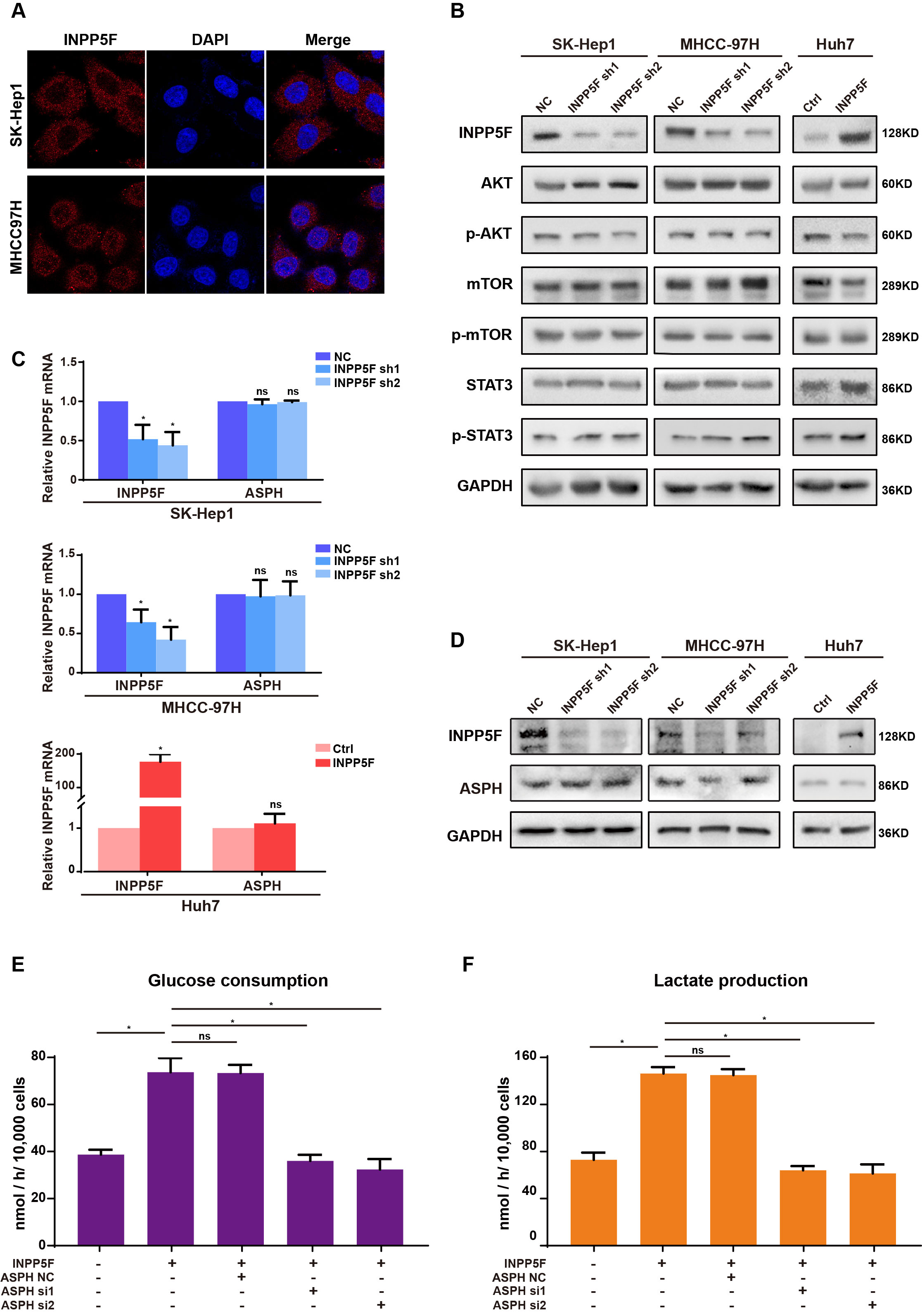


**Figure. S4** **INPP5F promotes aerobic glycolysis via interacting with cytoplasmic ASPH.** (A) Localization of INPP5F was determined by immunofluorescence. (B) The effect of INPP5F on AKT-mTOR and STAT3 was detected by western blot. (C) QRT-PCR and (D) western blot was performed to measure the regulation of ASPH by INPP5F. (E, F) Huh 7 cells overexpressing INPP5F were transfected with ASPH siRNA for 24 h. Glucose consumption and lactate production of cells with indicated treatments. Data is presented as means ± standard error for three independent experiments, *P < 0.05, ns: not significant.


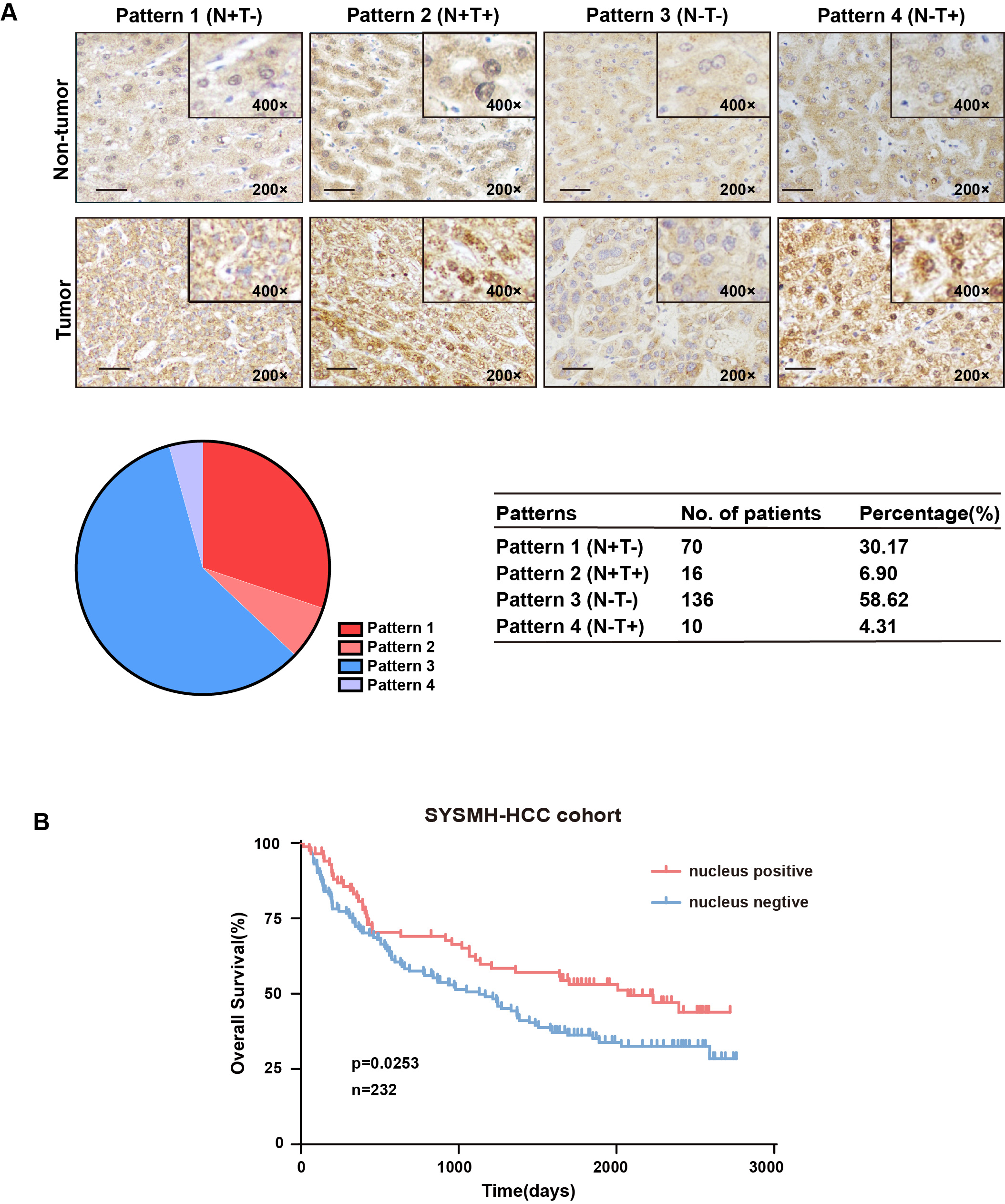
**Figure. S5** **Different sub-cellular expression patterns of INPP5F in HCC.** (A) The expression of INPP5F in HCC and adjacent tissues were detected by IHC. According to positive or negative nuclear staining in adjacent non-tumor(N) and tumor(T) tissues, patients were divided into four patterns: Pattern 1 (N+T-), Pattern 2 (N+T+), Pattern 3 (N-T-), and Pattern 4 (N-T+). Scale bar: 100 μm (up). Graphs showed the number of patients and the percentage of each pattern (down). (B) The clinical signification of nuclear INPP5F expression in adjacent tissues was evaluated in SYSMH-HCC cohort by Kaplan-Meier survival analyses. Scale bar: 100 um.


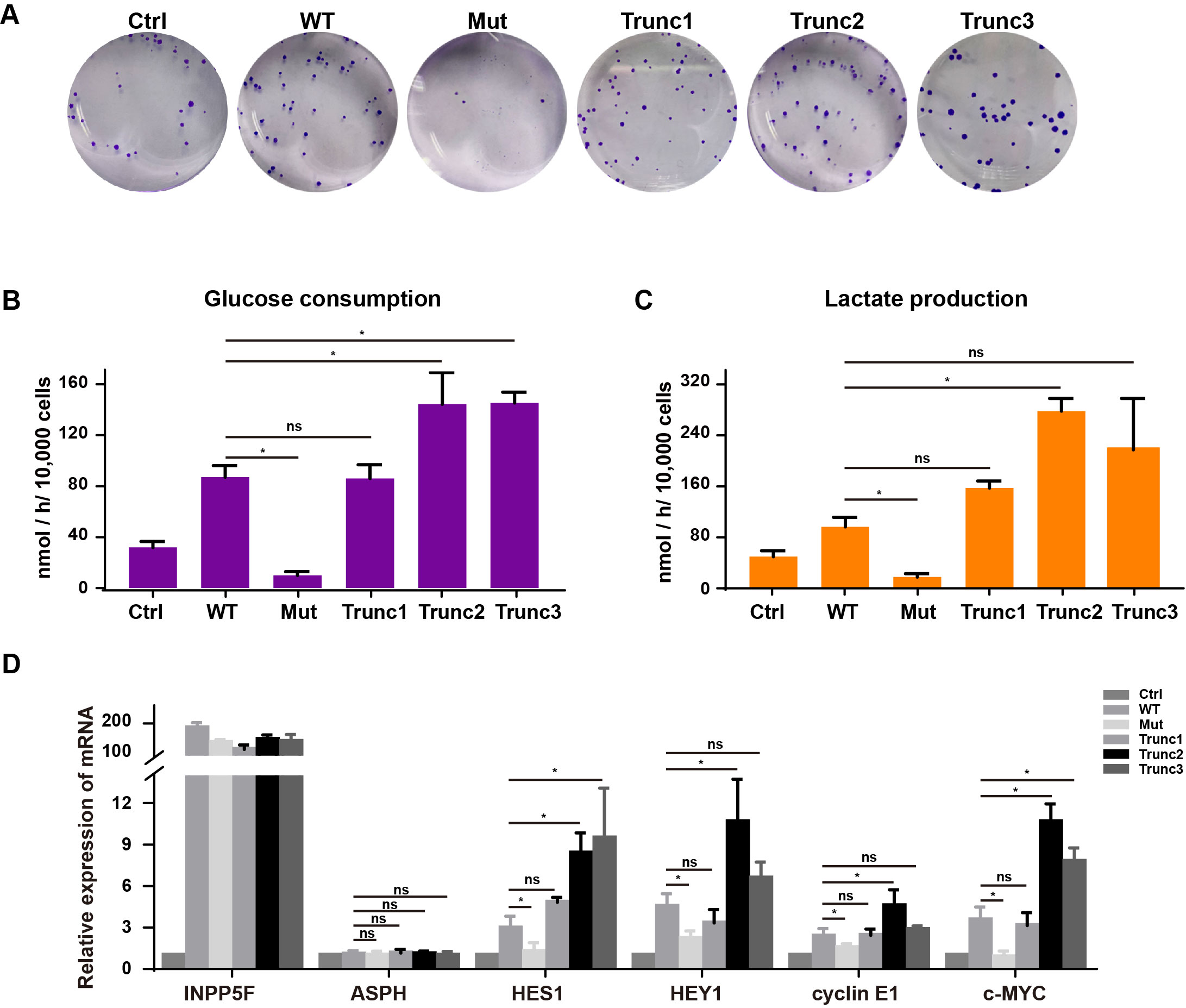
**Figure. S6 The NESs and NLSs of INPP5F affects its oncogenic activity by regulating sub-cellular location.** (A) The colony formation, (B) glucose consumption, (C) lactate production and (D) the mRNA expression of INPP5F-related downstream molecules of Huh7 cells which transfected with wild type (WT), NESs mutant or the three truncations. Data is presented as means ± standard error for three independent experiments, *P < 0.05, ns: not significant.


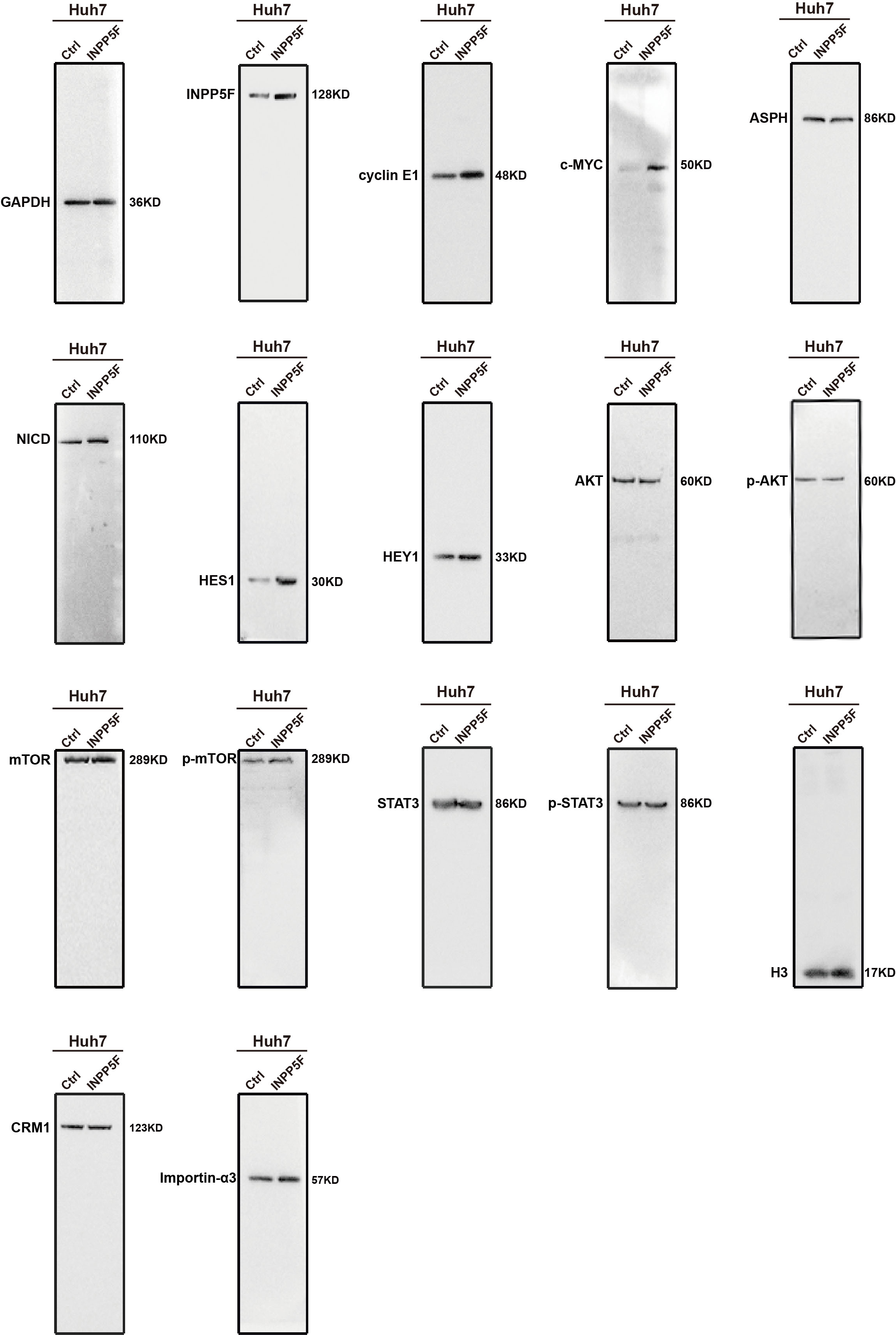


**Figure. S7 Full western blot image to confirm the molecular weight and specificity of the antibodies.**

**Supplementary Methods and Materials**

**Cell culture and transfection**

All cell lines were purchased from the Cell Resource Center, Chinese Academy of Science Committee (Shanghai, China). HCC cell lines SK-Hep1, MHCC-97H, PLC/PRF/5, HepG2, and Huh7 were cultured in DMEM supplemented with 10% fetal bovine serum and 1% antibiotics. Immortalize hepatic cell line LO2 and HCC cell line SMMC-7721 were cultured in RPMI-1640 containing 10% fetal bovine serum and 1% antibiotics (all reagents from Thermo Fisher Scientific, MA, USA). All cultures were maintained in a humidified 5% CO2 incubator at 37 °C.

Lentivirus constructed from INPP5F shRNAs (Genechem company, Shanghai, China) were transfected into the SK-Hep1 and MHCC-97H cells and then selected by 2 μg/mL puromycin for 2 weeks to establish stable cells. The INPP5F open reading frame was cloned into the plasmids pcDNA3.1 (GENEray, Shanghai, China). INPP5F overexpression or control plasmids were transfected into Huh7 cells by ViaFect Transfection Reagent (Promega, WI, USA). INPP5F and ASPH siRNAs were purchased from GenePharma company (Shanghai, China) and transfected into HCC cells by lipofectamine 3000 (Thermo Fisher Scientific, MA, USA) according to the manufacturer's protocol. Sequences of these siRNAs are listed in Table S3.

**Table S3 The si-RNA sequence list**

| INPP5F | Si-RNA-#1 | Forward, 5′- GGAGAUUACUUGAAGAGUUTT-3′ |
| --- | --- | --- |
|  |  | Reverse, 5′- AACUCUUCAAGUAAUCUCCTT -3 |
|  | Si-RNA-#2 | Forward, 5′- CCACCUGUGUAGAUGAUAUTT-3′ |
|  |  | Reverse, 5′- AUAUCAUCUACACAGGUGGTT-3′ |
| ASPH | Si-RNA-#3 | Forward, 5′- CACAGGAGAACCACAACAATT-3′ |
|  |  | Reverse, 5′- UUGUUGUGGUUCUCCUGUGTT-3′ |
|  | Si-RNA-#4 | Forward, 5′- CAGACAGGCAACAAUUUCUTT-3′, |
|  |  | Reverse, 5′- AGAAAUUGUUGCCUGUCUGTT-3’ |

**Quantitative real-time PCR (QRT-PCR) and RNA-Sequence (RNA-Seq) analysis**

Total RNA was extracted from HCC cell lines, fresh HCC and the para-tumor tissues by Trizol (Takara Bio, Shiga, Japan), and then reverse transcribed into cDNA using a PrimeScript™ RT reagent kit (Takara) according to the manufacturer’s protocols. The expression levels of mRNA were determined with QRT-PCR using SYBR green (Takara). The reactions were performed in triplicate. The primers used in QRT-PCR assays were listed in Table S4. SK-Hep1 cells was stably transfected with negative control or INPP5F shRNA. RNA-Seq analysis of total RNA extracted from these cells was performed by GENE DENOVO company (Guangzhou, China).

**Table S4** The primer sequence list

| INPP5F | Forward | 5′-CACATGCGCTTCCTCATCAC-3′ |
| --- | --- | --- |
|  | Reverse | 5′-CCAGCGGCGTTTTGTCATAG-3′ |
| GAPDH | Forward | 5′-ATTCCACCCATGGCAAATTCC-3′ |
|  | Reverse | 5′-GGGCAGAGATGATGACCCTT-3′. |
| Cyclin E1 | Forward | 5′-TGTCCTGGATGTTGACTGCC-3′ |
|  | Reverse | 5′-TCAGTTTTGAGCTCCCCGTC-3′. |
| c-MYC | Forward | 5′-CATCAGCACAACTACGCAGC-3′ |
|  | Reverse | 5′-CGTTGTGTGTTCGCCTCTTG-3′. |
| ASPH | Forward | 5′-CTACGTGGAGCCATCGAGAC-3′ |
|  | Reverse | 5′-ATCCCACGCCAAGGTCATTT-3′. |
| HES1 | Forward | 5′-GGCGGACATTCTGGAAATGAC-3′ |
|  | Reverse | 5′-ACCTCGGTATTAACGCCCTC-3′. |
| HEY1 | Forward | 5′-TGCAGATGACCGTGGATCAC-3′ |
|  | Reverse | 5′-GCTGGGAAGCGTAGTTGTTG-3′. |
| HK2 | Forward | 5′-TGCCACCAGACTAAACTAGACG-3′ |
|  | Reverse | 5′-CCCGTGCCCACAATGAGAC-3′ |
| HIF1A | Forward | 5′-GAACGTCGAAAAGAAAAGTCTCG-3′ |
|  | Reverse | 5′-CCTTATCAAGATGCGAACTCACA-3′ |
| GLUT1 | Forward | 5′-AAACCGGCGAGGAAAGGATG-3′ |
|  | Reverse | 5′-TCGAACCAAGACTCCTGTGG-3′ |
| GLUT3 | Forward | 5′-GCTGGGCATCGTTGTTGGA-3′ |
|  | Reverse | 5′-GCACTTTGTAGGATAGCAGGAAG-3′ |

**Western blotting**

Total protein was extracted with lysis buffer (Beyotime, Shanghai, China) containing protease inhibitors and subjected to quantitative determination. Nuclear protein extracts were performed with Nuclear and Cytoplasmic Extraction Kit (Beyotime) according to the manufacturer’s protocols. The proteins were separated on SDS-PAGE gels and then transferred onto PVDF membranes (Millipore, MA, USA). Subsequently, the membranes were blocked with 5% nonfat dry milk and incubated with specific primary antibodies. After overnight incubation at 4 °C and washed with TBST, the blotted membranes were incubated with HRP-conjugated secondary antibody at 1:10000 dilutions for 1h at room temperature. Immunoblots were probed with ECL detection reagent (Millipore) according to standard protocols. The detailed information of antibodies was shown in Table S5.

**Table S5 The list of antibodies for western blot**

| Anti-Homo sapiens primary antibodies | | | |
| --- | --- | --- | --- |
| Antibodies | Dilution | Identifiter | Source |
| Mouse anti-human GAPDH | 1: 5000 | 60004-1-Ig | Proteintech, IL, USA |
| Mouse anti-human INPP5F | 1: 500 | ab236391 | Abcam, Cambridge, UK |
| Rabbit anti-human Cyclin E1 | 1: 500 | 11554-1-AP | Proteintech, IL, USA |
| Rabbit anti-human c-MYC | 1: 1000 | 10828-1-AP | Proteintech, IL, USA |
| Rabbit anti-human APSH | 1: 1000 | ab172475 | Abcam, Cambridge, UK |
| Rabbit anti-human NICD | 1: 1000 | #4147 | Cell Signaling Technology, MA, USA |
| Rabbit anti-human HES1 | 1: 1000 | #11988 | Cell Signaling Technology, MA, USA |
| Rabbit anti-human HEY1 | 1: 500 | A16110 | ABclonal, Wuhan, China |
| Mouse anti-human AKT | 1: 1000 | #2966 | Cell Signaling Technology, MA, USA |
| Rabbit anti-human phospho-AKT | 1: 1000 | #4060 | Cell Signaling Technology, MA, USA |
| Rabbit anti-human mTOR | 1: 1000 | #2983 | Cell Signaling Technology, MA, USA |
| Rabbit anti-human phospho-mTOR | 1: 1000 | #5536 | Cell Signaling Technology, MA, USA |
| Rabbit anti-human STAT3 | 1: 2000 | #4904 | Cell Signaling Technology, MA, USA |
| Rabbit anti-human phosphor- STAT3 | 1: 2000 | #9145 | Cell Signaling Technology, MA, USA |
| Mouse anti-human FLAG-tag | 1: 2000 | AE005 | ABclonal, Wuhan, China |
| Mouse anti-human HA-tag | 1: 2000 | AE008 | ABclonal, Wuhan, China |
| Histone H3 | 1: 1000 | AH433 | Beyotime, Shanghai, China |
| CRM1 | 1: 500 | sc-74454 | Santa Cruz Biotechnology, TX USA |
| Importin-α | 1: 1000 | ER1912-10 | HuaBio,HangZhou, China |
| Secondary antibodies for Western blot | | | |
| Goat anti-mouse | 1:10,000 | sc-2005 | Santa Cruz Biotechnology, TX USA |
| Goat anti-rabbit | 1:10,000 | sc-2004 | Santa Cruz Biotechnology, TX USA |

**Colony formation**

Cells were collected and seeded in 6-well plates at a density of 2.0 × 10^3^ per well, and then incubated for 14 days. Colonies were fixed with methanol, stained with 0.1% crystal violet and counted.

**Ethynyl-20-deoxyuridine (EdU) Incorporation Assay**

EdU Incorporation assay was performed with EdU assay kit (Ribobio, Guangzhou, China). Cells were culture in 96-well plates and incubated for 48 h. Then 50 μM of EdU was added to each well and cultured for additional 2 h. The cells were fixed with 4% formaldehyde for 15 min and treated with 0.5% Triton X-100 for 20 min. After washing with PBS, 100 μl of 1 × Apollo reaction cocktail was added and incubated for 30 min. After staining with 100 μl of Hoechst 33342 for 30 min, the cells were visualized under EVOS cell image system.

**Cell Cycle Analysis**

HCC cells were gathered, washed with PBS twice, fixed in 75% ice-cold alcohol overnight, and then incubated with PBS containing propidium iodide (PI, 10 μg/mL) and RNase A (0.5 mg/mL). The cells were analyzed with a flow cytometer.

**Immunofluorescence**

Cells were fixed with 4% paraformaldehyde for 20 min and permeabilized in 0.05% Triton X-100 (Solarbio, Beijing, China) for 5 min at room temperature. After being blocked 2% BSA for one hour, the cells were subsequently incubated with primary antibody at 4°C overnight. Alexa Fluor secondary antibodies (1:800, ab150079 or ab150077, Abcam) was incubated for 1 h at room temperature. The nucleus was stained by DAPI (Solarbio, Beijing, China). Images were obtained by laser scanning confocal microscopy (LSM710 or LSM780, Zeiss). The primary antibodies were showed as followed: Rabbit anti-human INPP5F (1:100; no. PA5-21562, Invitrogen) and Mouse anti-human CRM1(1:200 sc-74454, Santa Cruz Biotechnology).
